# The Ti Plasmid‐Encoded VirJ Functions as a Lysyl‐Phosphatidylglycerol Hydrolase in *Agrobacterium tumefaciens*

**DOI:** 10.1111/mmi.70061

**Published:** 2026-03-16

**Authors:** Britta Lotz, Lina Brodskaia, Christiane Fritz, Stefanie Hebecker, Maike K. Tauchert, Jennifer Breisch, Dieter Jahn, Franz Narberhaus, Jürgen Moser, Meriyem Aktas

**Affiliations:** ^1^ Institute for Microbiology Technische Universität Braunschweig Braunschweig Germany; ^2^ Microbial Biology Ruhr‐Universität Bochum Bochum Germany

**Keywords:** AcvB, *agrobacterium*, lipid hydrolase, lysyl‐phosphatidylglycerol, phospholipids, VirJ

## Abstract

*Agrobacterium tumefaciens* delivers oncogenic transfer DNA (T‐DNA) into plants via a type IV secretion system (T4SS). This process requires virulence factors from the tumor‐inducing (Ti) plasmid and chromosomal genes such as *acvB*. We previously identified AcvB as a lysyl‐phosphatidylglycerol (L‐PG) hydrolase. Loss of AcvB in the nopaline‐type strain C58 increases membrane L‐PG levels, thereby compromising T‐DNA transfer. Interestingly, octopine‐type strains harbor an additional truncated *acvB* homologue, *virJ*, on the Ti plasmid. The established L‐PG hydrolase function of AcvB and its similarity to VirJ motivated us to investigate the enzymatic function of VirJ. Purified recombinant VirJ hydrolyzed L‐PG to phosphatidylglycerol and lysine. Transient *virJ* expression in an *acvB* deletion strain reduced elevated L‐PG levels, rescuing impaired growth under acidic conditions and defective T‐DNA transfer. These results provide the first direct evidence that VirJ functions as an L‐PG hydrolase and demonstrate that its enzymatic activity in maintaining L‐PG homeostasis is crucial for T‐DNA transfer. Importantly, elevated L‐PG levels did not disrupt T4SS assembly but likely compromised its functionality once formed. In contrast, L‐PG excess did not affect the conjugative RP4 T4SS or the type VI secretion system. Together, these findings highlight a secretion system‐specific reliance on membrane lipid composition.

## Graphical abstract

We identify the long‐known virulence factor VirJ as a lysyl‐phosphatidylglycerol (L‐PG) hydrolase. Like the chromosomally encoded virulence factor AcvB, it prevents excessive accumulation of L‐PG in the membrane, thereby maintaining efficient T‐DNA transfer via the Type IV secretion system (T4SS) in *Agrobacterium tumefaciens* .

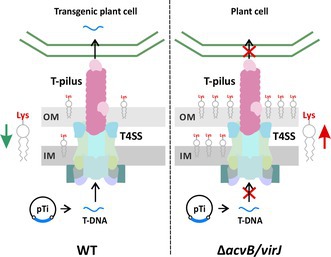

## 1 Introduction

*Agrobacterium tumefaciens* is a remarkable plant pathogen that is well known for its ability to transfer oncogenic DNA into plant cells, leading to the development of crown gall disease. The transfer‐DNA (T‐DNA) is located on the tumor‐inducing (Ti) plasmid, which also carries the essential genetic elements required for its transfer into plant cells (McCullen and Binns 2006; Brown et al. 2023). Central to this process are the virulence (*vir*) genes, including the *virB* operon and *virD4*, which encode components of a type IV secretion system (T4SS) that facilitates T‐DNA transfer (Li and Christie 2018). Transcription of these *vir* genes is regulated by the VirA/VirG two‐component regulatory system, which is activated by phenolic plant signal molecules such as acetosyringone and acidic conditions (Winans 1992; Lee et al. 1995; Brencic and Winans 2005). In addition to the well‐known *vir* genes, several chromosomal genes (*chv*) have also been identified as crucial for tumorigenesis, but their specific functions remain poorly understood (Douglas et al. 1985; Kalogeraki and Winans 1995; Goodner et al. 2001; Suzuki et al. 2001; Nester 2014). Among these genes, the highly conserved *acvB* gene was identified three decades ago as essential for T‐DNA transfer (Kalogeraki and Winans 1995). What distinguishes *acvB* from other *chv* genes is the presence of a homolog, *virJ*, which is found exclusively on octopine‐type Ti plasmids (Kalogeraki and Winans 1995; Pan et al. 1995; Weisberg et al. 2023). As a result, octopine‐type *A. tumefaciens* strains encode both the chromosomal *acvB* gene and the plasmid‐borne *virJ*, whereas nopaline‐type strains, such as C58, carry only *acvB*.

**FIGURE 1:**
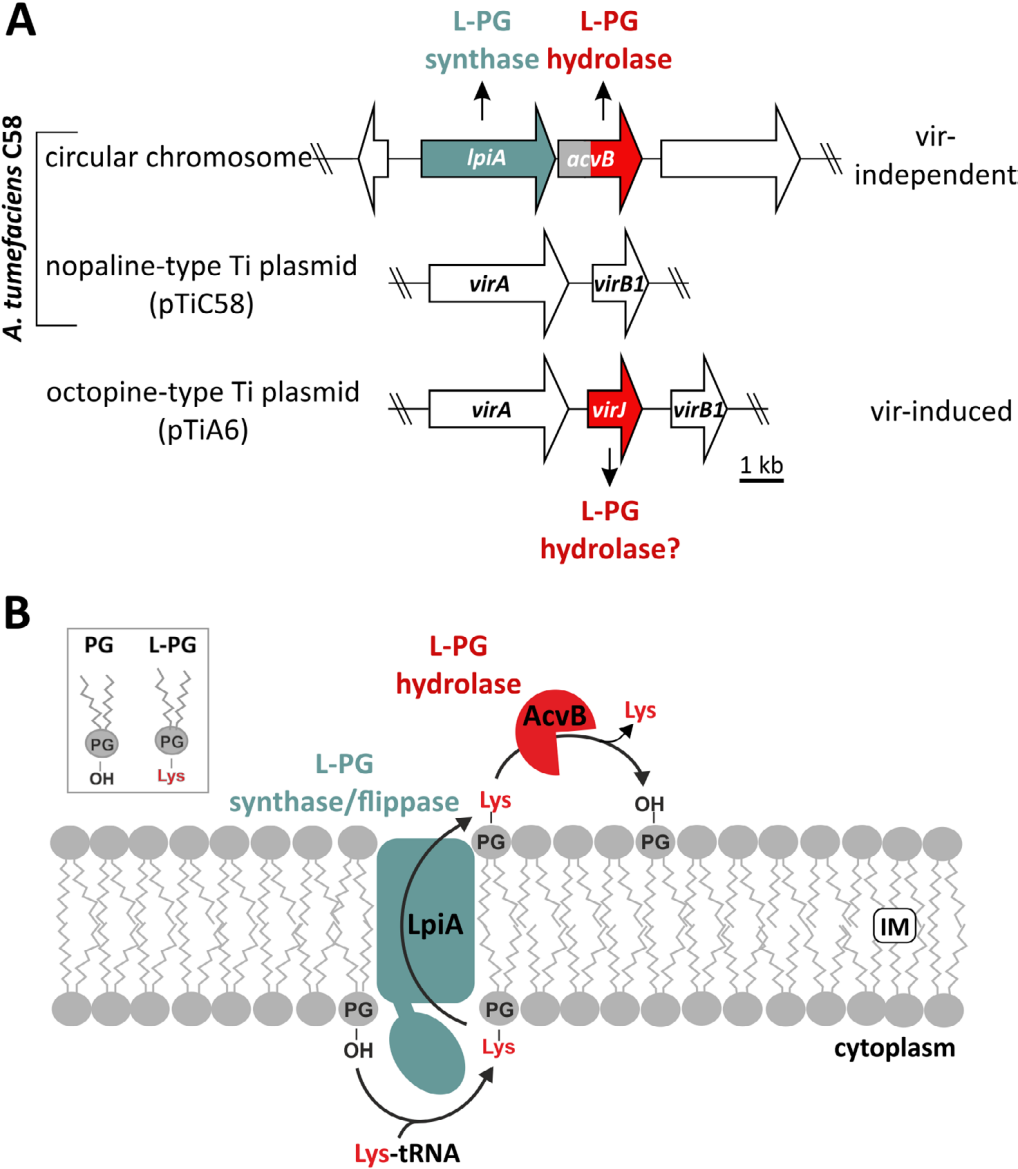
Genetic organization of *acvB* and *virJ* and the molecular function of the respective gene products in *Agrobacterium* strains. (A) The *acvB* gene is located on the circular chromosome of all *Agrobacterium* strains. Upstream of *acvB* is the *lpiA* gene, which encodes a bifunctional L‐PG synthase and flippase. The *acvB* homolog, *virJ*, is encoded exclusively on octopine‐type Ti plasmids, located between *virA* and the *virB* operon. Expression of *virJ* is regulated by the VirA/VirG system (vir‐induced). (B) The LpiA enzyme synthesizes L‐PG from PG and lysyl‐tRNA at the inner cytoplasmic membrane. L‐PG is subsequently translocated to the outer leaflet of the inner membrane. AcvB catalyzes the cleavage of L‐PG into PG and lysine.

On the Ti plasmid, the *virJ* gene is situated between the *virA* and *virB* genes, and its expression is regulated by the VirA/VirG system (Lin et al. 2014). In contrast, the *acvB* gene is located on the circular chromosome, forming an operon with *lpiA* (low pH inducible protein A), which encodes a lysyl‐phosphatidylglycerol (L‐PG) synthase (Figure 1A) (Vinuesa et al. 2003; Groenewold et al. 2019). Unlike *virJ*, the expression of *acvB* is independent of the VirA/VirG system.

Since their discovery as virulence factors, several possible roles for AcvB and VirJ in T‐DNA transfer have been proposed. One hypothesis suggests that AcvB and VirJ may facilitate T‐strand binding, possibly via effector proteins, to mediate T‐DNA transfer to plant cells (Kang et al. 1994; Kalogeraki and Winans 1995; Pantoja et al. 2002). However, this interaction between AcvB/VirJ and the T‐strand was later questioned by subsequent research (Cascales and Christie 2003). An alternative hypothesis suggests that AcvB and VirJ may act as periplasmic chaperones (Atmakuri et al. 2003; Cascales and Christie 2003). Despite these efforts, the precise functions of VirJ and AcvB in T‐DNA transfer remained unresolved. This mystery began to unravel with the recent biochemical characterization of AcvB, which identified its role in membrane lipid homeostasis as an L‐PG hydrolase. The periplasmic AcvB operates in tandem with the L‐PG synthase (LpiA) (Figure 1B), which produces L‐PG by transferring a lysyl moiety from a lysyl‐tRNA to phosphatidylglycerol (PG). Subsequently, the transmembrane protein LpiA facilitates the flipping of the modified phospholipid to the periplasmic side of the cytoplasmic membrane (Vinuesa et al. 2003; Roy 2009). Notably, acidic conditions promote the accumulation of L‐PG (Groenewold et al. 2019). At the periplasmic face of the inner membrane, AcvB hydrolyzes L‐PG to PG, releasing lysine, thereby maintaining the balance between these two membrane lipid species (Groenewold et al. 2019).

**FIGURE 2:**
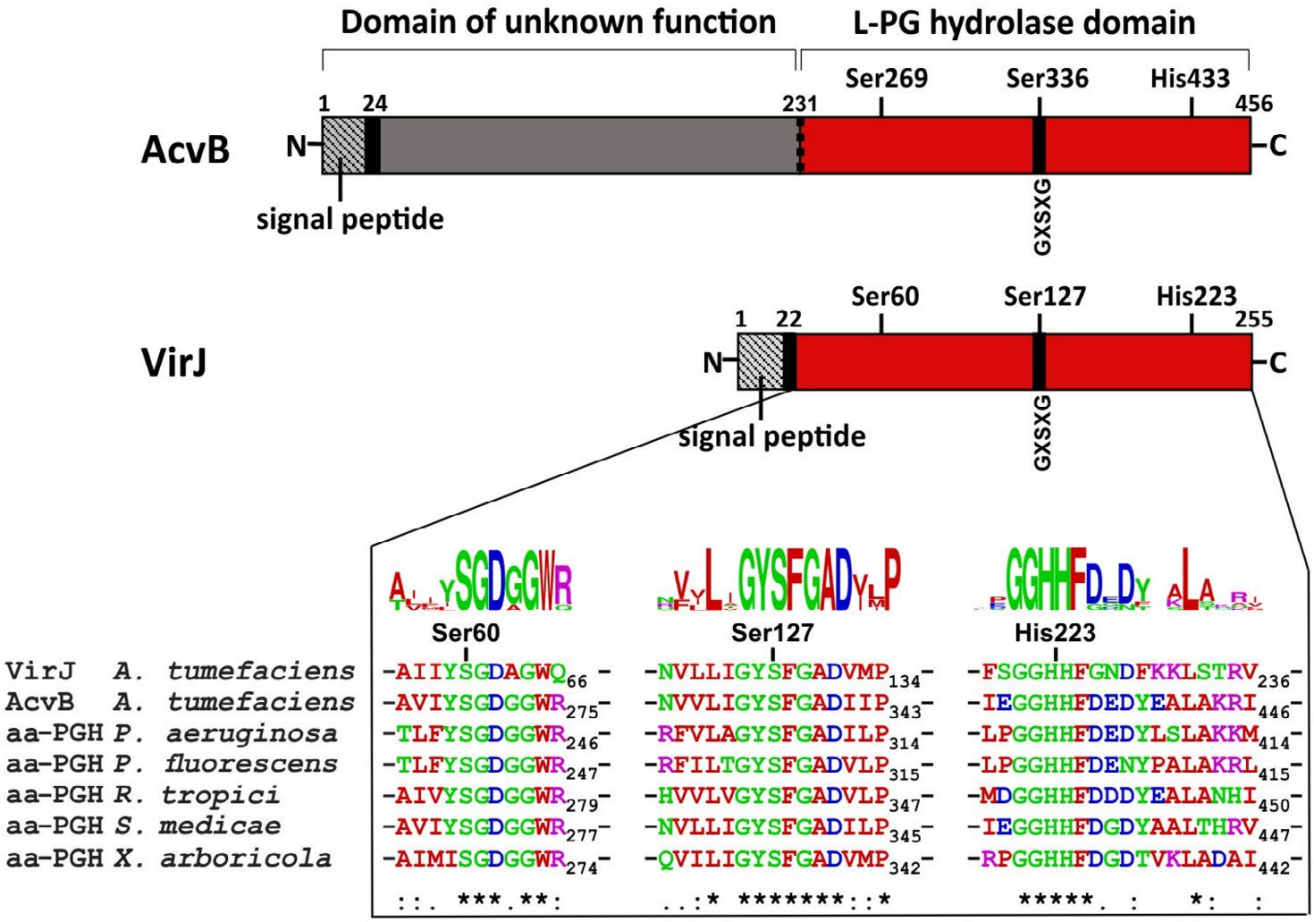
Postulated domain structure of *A. tumefaciens* AcvB and VirJ and partial sequence alignment of VirJ orthologs. Gene names are given as follows. *Agrobacterium tumefaciens* , AAF77160.1; *Agrobacterium tumefaciens* (or *Agrobacterium fabrum* str. C58), AAK88253.1; *Pseudomonas aeruginosa* PAO1, AAG04308.1; *Pseudomonas fluorescens* , AUM68555.1; *Rhizobium tropici* CIAT 899, AAN52238.1; *Sinorhizobium medicae* WSM419, AAP21142.1; *Xanthomonas arboricola* pv. *fragariae*, SOU12047.1. The conservation pattern across three distinct regions is represented as a sequence logo, with conserved residues marked by an asterisk and partially conserved residues indicated by a colon or period. Amino acid positions in VirJ targeted for site‐directed mutagenesis are highlighted. aa‐PGH, Aminoacyl phosphatidylglycerol hydrolase.

AcvB (~460 amino acids) is nearly twice the size of VirJ (~250 amino acids) and consists of two distinct domains (Figure 2). The C‐terminal catalytic domain of AcvB shares ~50% sequence identity with VirJ (Kalogeraki and Winans 1995). Notably, only this C‐terminal domain is required for the enzymatic activity of AcvB and for its crucial role in T‐DNA transfer (Groenewold et al. 2019). In the nopaline‐type *A. tumefaciens* C58 strain lacking a *virJ* homolog, *acvB* deletion leads to increased L‐PG levels. This altered membrane lipid composition may disrupt the T4SS and impair T‐DNA transfer to the host. However, the underlying molecular mechanism remains unknown (Groenewold et al. 2019). In contrast, in octopine‐type strains deficient in *acvB*, the virulence plasmid‐encoded VirJ protein can functionally compensate for the absence of AcvB (Kalogeraki and Winans 1995).

In *A. tumefaciens* and other proteobacteria, L‐PG is typically present at very low levels under acidic growth conditions (1%–2% of total membrane lipids) (Sohlenkamp et al. 2007; Groenewold et al. 2019). Gram‐positive bacteria can accumulate L‐PG to much higher proportions (up to 60% of total membrane lipids) (Peschel et al. 2001; Vásquez et al. 2025), where it plays a well‐established role in resistance to cationic antimicrobial peptides (CAMPs). The proposed mechanism is that increased levels of the cationic membrane lipid L‐PG reduce the net negative charge of the cytoplasmic membrane, thereby decreasing the affinity for CAMPs. In addition, L‐PG may also contribute to resistance against non‐cationic antimicrobial agents, although the underlying mechanisms are not yet fully understood (den Kamp et al. 1969; Haest et al. 1972; Roy 2009). The role of L‐PG homeostasis in Gram‐negative bacteria remains comparatively less explored. In *Rhizobium tropici* CIAT899, L‐PG production has been shown to enhance resistance to polymyxin B under acidic conditions, underscoring its importance in stress adaptation and survival in plant‐associated environments (Sohlenkamp et al. 2007).

In this study, we elucidate the enzymatic function of the proposed virulence factor VirJ and identify it as an L‐PG hydrolase. We show that VirJ‐mediated regulation of L‐PG levels in the bacterial membrane is essential for T‐DNA transfer via the T4SS. In contrast, the conjugative RP4 T4SS and the type VI secretion system of *A. tumefaciens* remain unaffected by the absence of L‐PG hydrolase activity. These findings underscore the importance of membrane lipid composition in modulating the function of distinct bacterial translocation systems.

## 2 Results

### 2.1 VirJ Is Homologous to the C‐Terminal Catalytic Domain of AcvB

In this study, we aimed to elucidate the biological function of VirJ. VirJ is half the size of AcvB, and both proteins have been shown to contain an N‐terminal signal sequence for periplasmic localization (Pan et al. 1995; Pantoja et al. 2002; Groenewold et al. 2019). Bioinformatic predictions using SignalP (Teufel et al. 2022) identified a potential signal sequence for VirJ, with a cleavage site after amino acid 22. Further sequence analysis using Clustal Omega (Sievers et al. 2011) revealed that VirJ shares 46% sequence identity with the C‐terminal half of AcvB. AcvB mediates L‐PG hydrolase activity via the key catalytic residues Ser269, Ser336, and His433 (Groenewold et al. 2019). The sequence comparison in Figure 2 indicates that these residues correspond to Ser60, Ser127, and His223 of VirJ. Based on these findings, we hypothesized that VirJ functions as a periplasmic L‐PG hydrolase.

VirJ was confirmed to localize in the periplasm via PhoA fusion assays. A plasmid (pTrc200_virJ‐phoA) encoding a VirJ signal sequence‐PhoA fusion was constructed and tested in *A. tumefaciens* C58. Periplasmic alkaline phosphatase activity was assessed by monitoring 5‐bromo‐4‐chloro‐3‐indolyl phosphate (BCIP) hydrolysis on YEB plates, with blue color development indicating periplasmic localization. Control plasmids were included, with pTrc200 encoding native PhoA as a positive control and PhoA without a signal sequence as a negative control (Figure S1).

### 2.2 L‐PG Hydrolase Activity of VirJ

**FIGURE 3:**
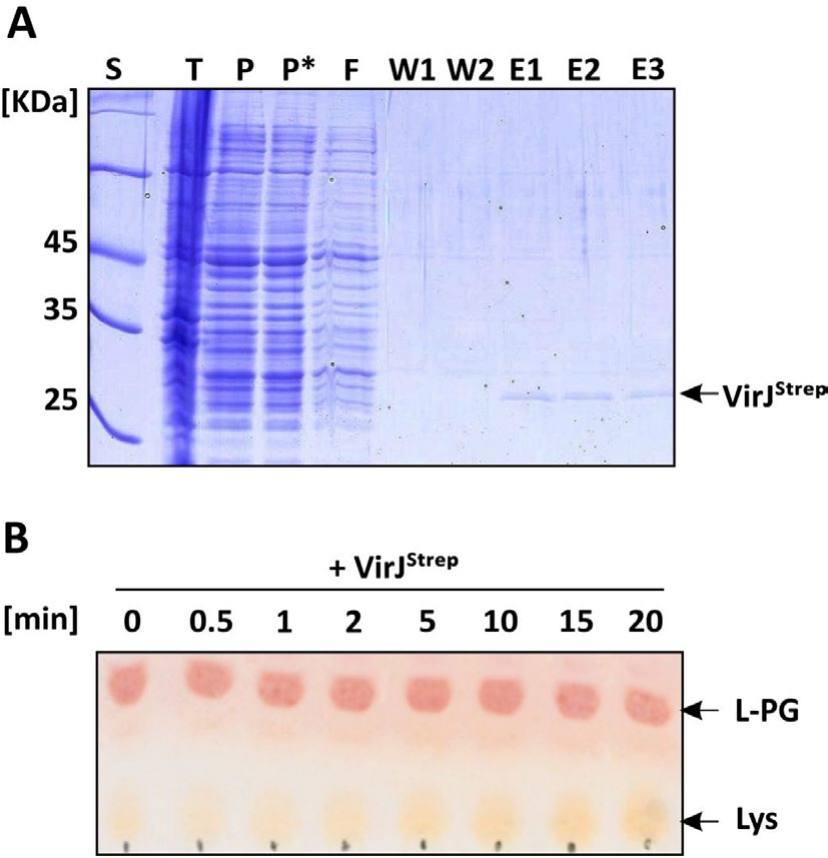
Purification and in vitro activity of VirJ. (A) Purification profile of C‐terminally Strep‐tagged VirJ from the periplasmic fraction following osmotic shock lysis. E1‐3, elution fractions; F, flow through; P*, periplasmic fraction with streptavidine; P, periplasmic fraction; T, total cell extract; W1‐2, washing fractions. (B) In vitro L‐PG hydrolysis activity of VirJ^Strep^. L‐PG was incubated with VirJ^Strep^ for 20 min, with samples collected at indicated time points and analyzed using 1D‐TLC. Amino groups were detected via ninhydrin staining. Note that commercial L‐PG is not completely pure and typically contains small amounts of free lysine. Data shown are representative of three independent experiments.

For biochemical characterization, a C‐terminally Strep‐tagged version of VirJ (from amino acid 23) containing an N‐terminal PelB leader sequence was overexpressed in *E. coli* and purified from the periplasmic fraction using affinity chromatography. Although purified in small quantities, the protein was obtained in a pure form (Figure 3A). The in vitro activity of VirJ^Strep^ was assessed using one‐dimensional thin‐layer chromatography (1D‐TLC). For this, the recombinant protein was incubated with commercially available L‐PG. After different time points, samples were collected and analyzed for liberation of lysine from the substrate L‐PG. Lysine release was detected by ninhydrin staining. As shown in Figure 3B, increasing lysine spot intensity over 20 min indicated L‐PG hydrolase activity of VirJ. Densitometric analysis revealed the release of 9.3 nmol lysine in the presence of approximately 20 nmol VirJ.

### 2.3 Episomal Expression of *virJ* Counteracts the Elevated L‐PG Levels in the *A. tumefaciens* C58 Δ*acvB* Mutant

**FIGURE 4:**
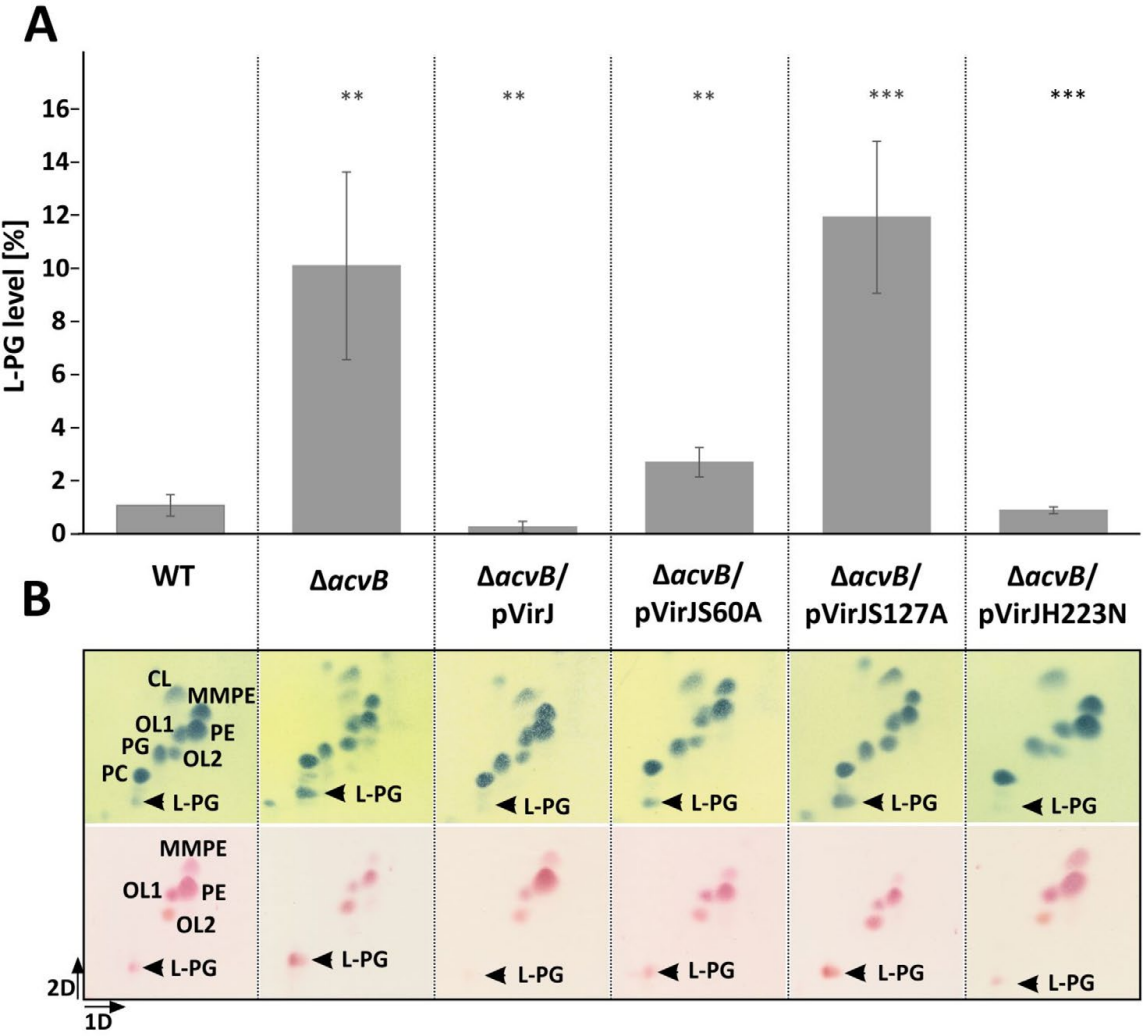
VirJ rescues the increased L‐PG accumulation in the C58 *acvB* mutant. The strains were cultivated 24 h in AB‐MES minimal medium at pH 5.5 with IPTG to induce *virJ* expression and total lipids were extracted and separated by 2D‐TLC using solvent system (1) for separation. (A) Relative amounts of L‐PG in different strains. L‐PG spot intensity after molybdatophosphoric acid staining was quantified using GelQuant and normalized relative to the total (dominant) lipid content (CL, MMPE, PE, OL1, OL2, PC, PG and L‐PG). Mean values for L‐PG content were calculated from the results of at least three independent experiments and error bars show standard errors of the means. Statistical analysis revealed *p* values of ≤ 0.01 (**), and *p* values of ≤ 0.001 (***). (B) Molybdatophosphoric acid (above) and ninhydrin staining (below) of the 2D‐TLC plates (representative examples from at least three independent experiments). L‐PG, lysyl‐PG; MMPE, monomethyl‐PE; OL1/2, ornithine lipids 1/2; PC, phosphatidylcholine; PE, phosphatidylethanolamine; PG, phosphatidylglycerol.

The nopaline‐type *A. tumefaciens* C58 strain contains the *acvB* gene but lacks the Ti plasmid‐encoded *virJ*. The C58 *acvB*‐mutant strain is characterized by elevated L‐PG levels (Groenewold et al. 2019). VirJ was episomally produced in this mutant strain using the IPTG‐inducible expression vector pTrc200. This complemented strain (Δ*acvB*/pVirJ) served as the system for investigating VirJ function under physiological conditions. The strain was cultured in AB‐MES medium at pH 5.5, with IPTG supplementation to induce *virJ* expression. Acidic conditions are known to promote L‐PG accumulation (Groenewold et al. 2019). In addition to the wild‐type VirJ, variants with substitutions in the putative catalytically relevant residues (Ser60, Ser127, and His223) (Figure 2) were constructed and analyzed accordingly (Δ*acvB*/pVirJS60A, Δ*acvB*/pVirJS127A and Δ*acvB*/pVirJH223N). The L‐PG levels of all strains were assessed using 2‐dimensional thin‐layer chromatography (2D‐TLC) of the extracted total lipids. Molybdenum blue staining was employed to visualize the total phospholipid pattern, while ninhydrin staining was used specifically to detect L‐PG levels (Figure 4B). The relative L‐PG amount of the strains was quantified by densitometry of the molybdenum blue‐stained lipid spots (Figure 4A).

As previously reported, *A. tumefaciens* C58 contains L‐PG levels between 1% and 2%, which increases to approximately 10% in the *acvB* mutant (Figure 4 and (Groenewold et al. 2019)). Introduction of the *virJ* into the *acvB* mutant reduced the L‐PG level to around 0.2%, clearly demonstrating the in vivo L‐PG hydrolase activity of VirJ and its ability to compensate for the enzymatic activity of AcvB.

Like the wild‐type VirJ, the VirJH223N variant effectively reduced the elevated L‐PG levels in the *acvB* mutant (Figure 4). This was also observed for the H223A variant (data not shown), indicating that mutation of His223 does not abolish the L‐PG hydrolase activity of VirJ. In the VirJS60A‐producing *acvB* mutant, L‐PG levels were also reduced compared to the *acvB* mutant, though not to the same extent as with wild‐type VirJ, suggesting that the S60A mutation retains partial activity. Thus, Ser60 contributes to but is not essential for catalytic function. These observations broadly align with the corresponding residues in AcvB. In this case, mutation of His433 (corresponding to VirJ His223) results in intermediate activity, whereas substitution of S269 (corresponding to VirJ Ser60) leaves the protein fully active, suggesting that while the catalytic roles of these residues are generally conserved, their specific effects differ between VirJ and AcvB. By contrast, the high L‐PG level (12%) observed in the *acvB* mutant producing VirJS127A indicates that Ser127 is a key residue for the L‐PG hydrolase activity of VirJ, consistent with the corresponding Ser336 in AcvB required for catalysis (Groenewold et al. 2019).

To determine whether the accumulation of L‐PG in the absence of AcvB also influences other membrane lipids, we quantified the overall phospholipid composition in the same experiment. As shown in Figure S2, the total phospholipid profiles of the tested strains were largely comparable. Strains exhibiting elevated L‐PG levels (Δ*acvB* and *acvB*/VirJS127A) showed only a slight increase in ornithine lipid 2 (OL2), accompanied by reduced phosphatidylethanolamine (PE) and monomethyl‐PE (MMPE) levels. These subtle changes suggest an adaptive lipid remodeling response that likely helps maintain membrane stability under conditions of disturbed L‐PG homeostasis.

### 2.4 VirJ Compensates the Growth Defect and Recovers T4SS Functionality in the C58 Δ*acvB* Mutant

**FIGURE 5:**
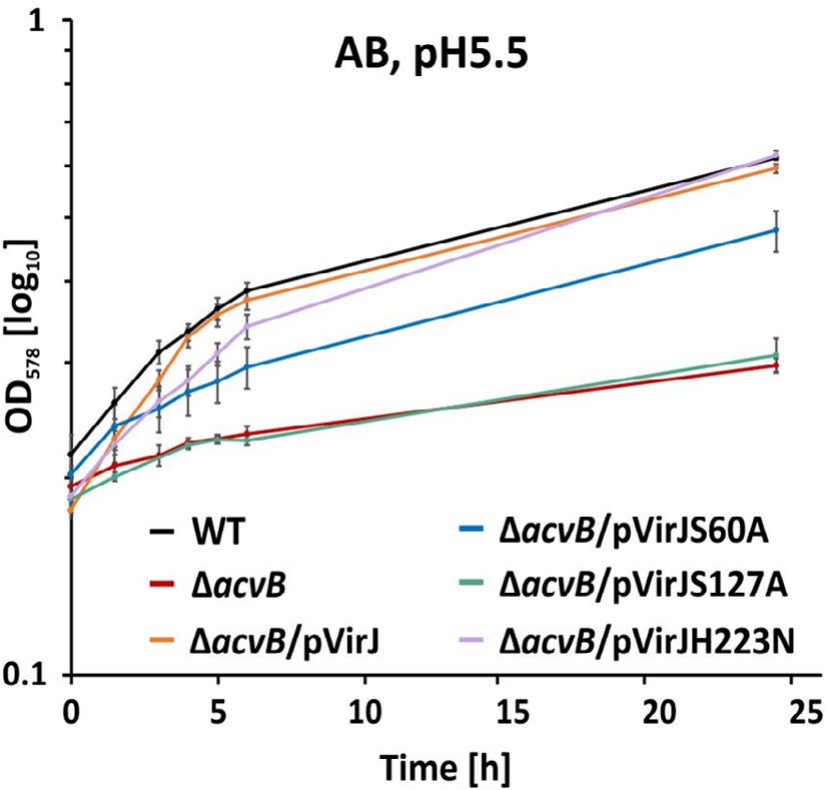
VirJ rescues the growth defect under acidic conditions in the C58 Δ*acvB* mutant. Growth behavior of different *A. tumefaciens* strains complemented with different VirJ variants. VirJ was episomally produced from pTrc200. Indicated strains were cultivated in AB‐MES medium (pH 5.5) containing IPTG to induce gene expression and OD_578_ of the cultures was monitored over 24 h. Mean values for the OD_578_ at 0, 1.5, 3, 4, 5, 6, and 24 h were calculated from the results of three independent experiments and error bars show standard errors of the means.

As we previously demonstrated, the lack of *acvB* in the C58 strain induces a growth defect under acidic conditions (Groenewold et al. 2019). This growth defect can be complemented by episomal *virJ* expression to wild‐type levels (Figure 5). While the VirJ H223N variant compensated similarly to the wild‐type VirJ, the S60A variant only showed intermediate restoration of the growth defect. In contrast, the inactive VirJS127A variant was unable to compensate for the lack of AcvB (Figure 5), which is consistent with the lipid profiling results (Figure 4). Collectively, these findings demonstrate that VirJ can functionally compensate for the absence of AcvB, with Ser127 playing the most important role among the three selected residues.

**FIGURE 6:**
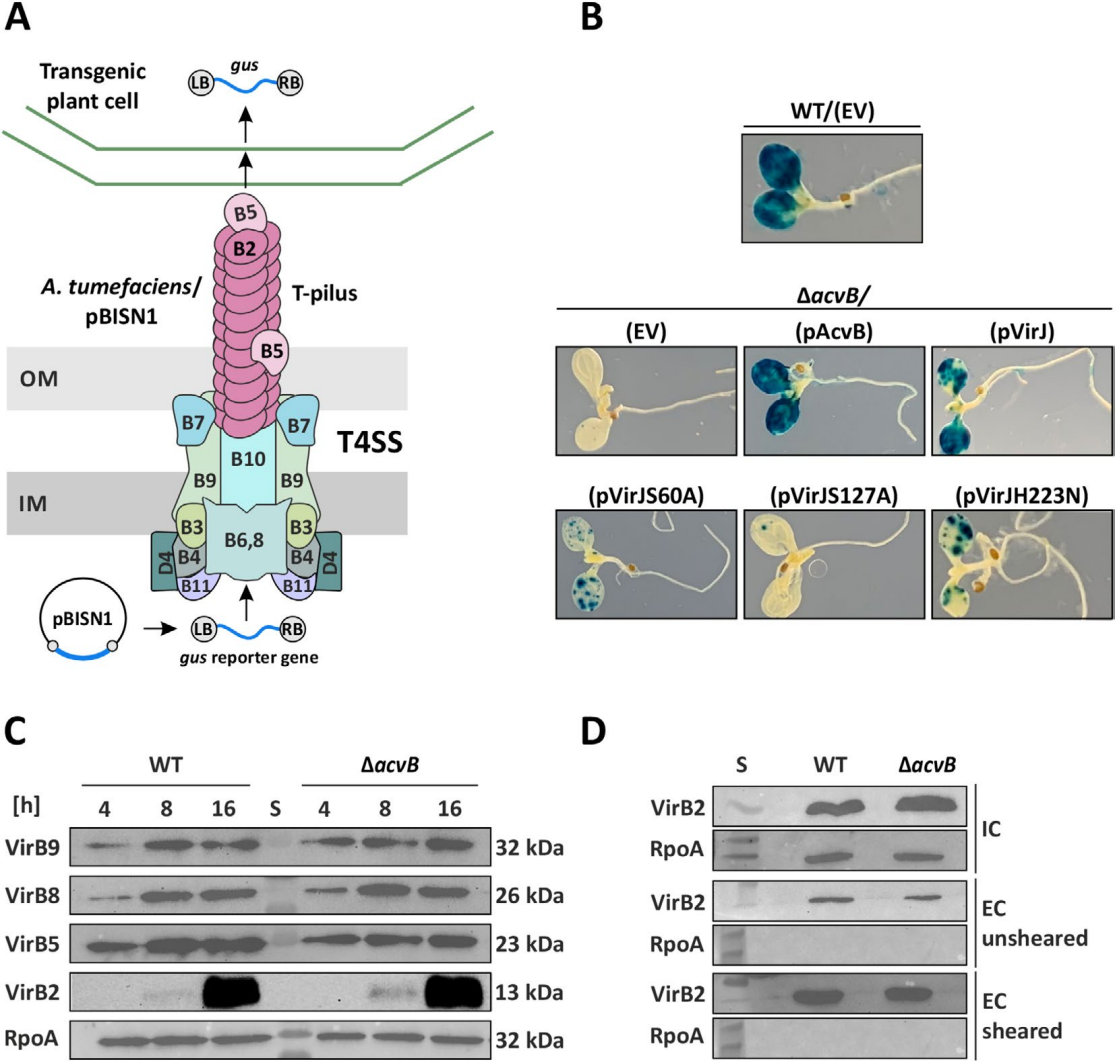
VirJ restores type IV secretion system (T4SS) function in the C58 Δ*acvB* mutant. (A) T4SS of *A. tumefaciens* adapted from (McCullen and Binns 2006) and GUS transient transformation assay. (B) Transient transformation of *Arabidopsis* seedlings by *A. tumefaciens* . Seven‐day‐old *Arabidopsis thaliana efr‐1* seedlings were inoculated with wild‐type (WT) *A. tumefaciens* or the Δ*acvB‐*mutant strain, both carrying the GUS reporter plasmid pBISN1 and the indicated pTrc200‐derived constructs. Three days of post‐inoculation, GUS activity was visualized by histochemical staining to assess transient expression efficiency. EV: Empty vector (pTrc200); LB, left border; RB, right border. Representative seedlings from each treatment are shown. Data are representative of three independent experiments, each using at least 30 seedlings. (C) Time‐course Western blot analysis of VirB proteins in virulence‐induced *A. tumefaciens* cells. At the indicated time points following acetosyringone induction, samples were collected and analyzed using VirB‐specific antibodies. (D) Detection of the extracellular T‐pilus. The major T‐pilus component, VirB2, was detected in T‐pilus‐induced culture supernatants (EC) from sheared or unsheared cells. Intracellular (IC) VirB2 is shown as a control. The cytoplasmic RpoA, protein, was used as internal loading control for signal quantification for cell extracts and to verify the absence of cellular contamination in the supernatant samples. The Western blots shown are representative of two independent experiments. S, protein standard.

This trend was also true for T‐DNA transfer efficiency. T‐DNA transfer of *A. tumefaciens* strains was analyzed using the *Agrobacterium*‐mediated transient *gus*‐expression assay AGROBEST with *Arabidopsis* seedlings (Figure 6A) and (Wu, Liu, et al. 2014; Groenewold et al. 2019; Wu and Lai 2022). As we previously demonstrated (Groenewold et al. 2019), the C58 *acvB* mutant strain was defect in T‐DNA transfer (Figure 6B). This defect could be restored to wild‐type levels by either episomal *acvB* or *virJ* expression. While the VirJ S60A and H223N variants showed intermediate compensation of the T‐DNA transfer defect, the inactive S127A variant was unable to rescue the T‐DNA transfer defect (Figure 6B and Figure S3). Accordingly, T‐DNA transfer efficiency correlates with the lipid profiles of the different *A. tumefaciens* strains. Specifically, the overabundance of L‐PG in L‐PG hydrolase‐deficient strains is detrimental to T‐DNA transfer.

Collectively, these data clearly demonstrate that VirJ functions as an L‐PG hydrolase that helps to maintain the balance between PG and L‐PG in the membrane. The absence of both AcvB and VirJ disrupts this lipid equilibrium, potentially compromising membrane integrity and function. Such an imbalance may impair the proper assembly or activity of the T4SS as suggested by our previous publication (Groenewold et al. 2019). The *A. tumefaciens* VirB/VirD4 T4SS consists of an envelope‐spanning translocation channel formed by the VirD4 coupling protein together with VirB1–VirB11, as well as an extracellular T‐pilus composed of the major subunit VirB2 and the minor component VirB5 (Figure 6A) (Lai et al. 2000; Christie 2001; Wu, Chen, and Lai 2014).

In our previous study, qualitative Western blot analysis revealed that elevated L‐PG accumulation in the *acvB* mutant does not prevent T4SS formation, as core components of the system were detected, albeit at slightly reduced levels of VirB8 and VirB5 after 18 h of virulence induction (Groenewold et al. 2019). However, this initial analysis lacked a quantitative comparison of VirB protein levels between the wild‐type and mutant strains. Therefore, to enable quantitative analysis, we performed a time‐course Western blot using samples collected at 4, 8, and 16 h following virulence induction. We examined the abundance of VirB8, VirB9, VirB5, and VirB2 in cell extracts from both strains, using RpoA as an internal loading control for signal quantification. The results revealed comparable levels of these VirB proteins in the *acvB* mutant and the wild‐type strain at all time points (Figure 6C), suggesting that the previously observed minor differences likely resulted from the qualitative nature of the initial analysis. Importantly, these findings support our previous conclusion that elevated L‐PG levels in the *acvB* mutant do not impair VirB/VirD4 system production (Groenewold et al. 2019).

The T‐pilus plays a critical role in T‐DNA transfer, especially in transient transformation efficiency in unwounded *Arabidopsis* seedlings (Wu, Chen, and Lai 2014). T‐pilus biogenesis depends on the correct assembly and coordination of multiple VirB subcomplexes; all VirB proteins (VirB1–VirB11), but not the VirD4 coupling protein, have been shown to be essential for this process (Lai et al. 2000; Christie 2004).

To verify that the VirB/VirD4 system is properly assembled, we assessed T‐pilus formation by Western blot analysis of extracellular fractions, detecting the major subunit VirB2. *A. tumefaciens* cells induced for T‐pilus production were scraped from agar plates, resuspended in acidic phosphate buffer, and centrifuged to obtain the unsheared extracellular (EC) fraction, or subjected to shearing to obtain the sheared EC fraction enriched for T‐pili. Overall, the levels of extracellular VirB2 were similar in the wild‐type and the *acvB* mutant in both unsheared and sheared fractions, with higher levels of VirB2 detected in the sheared fraction (Figure 6D), indicating that the T4SS is correctly assembled. Thus, elevated L‐PG levels affect T4SS function after assembly rather than its biogenesis.

### 2.5 T6SS Function Remains Unaffected by Alterations in L‐PG Levels

**FIGURE 7:**
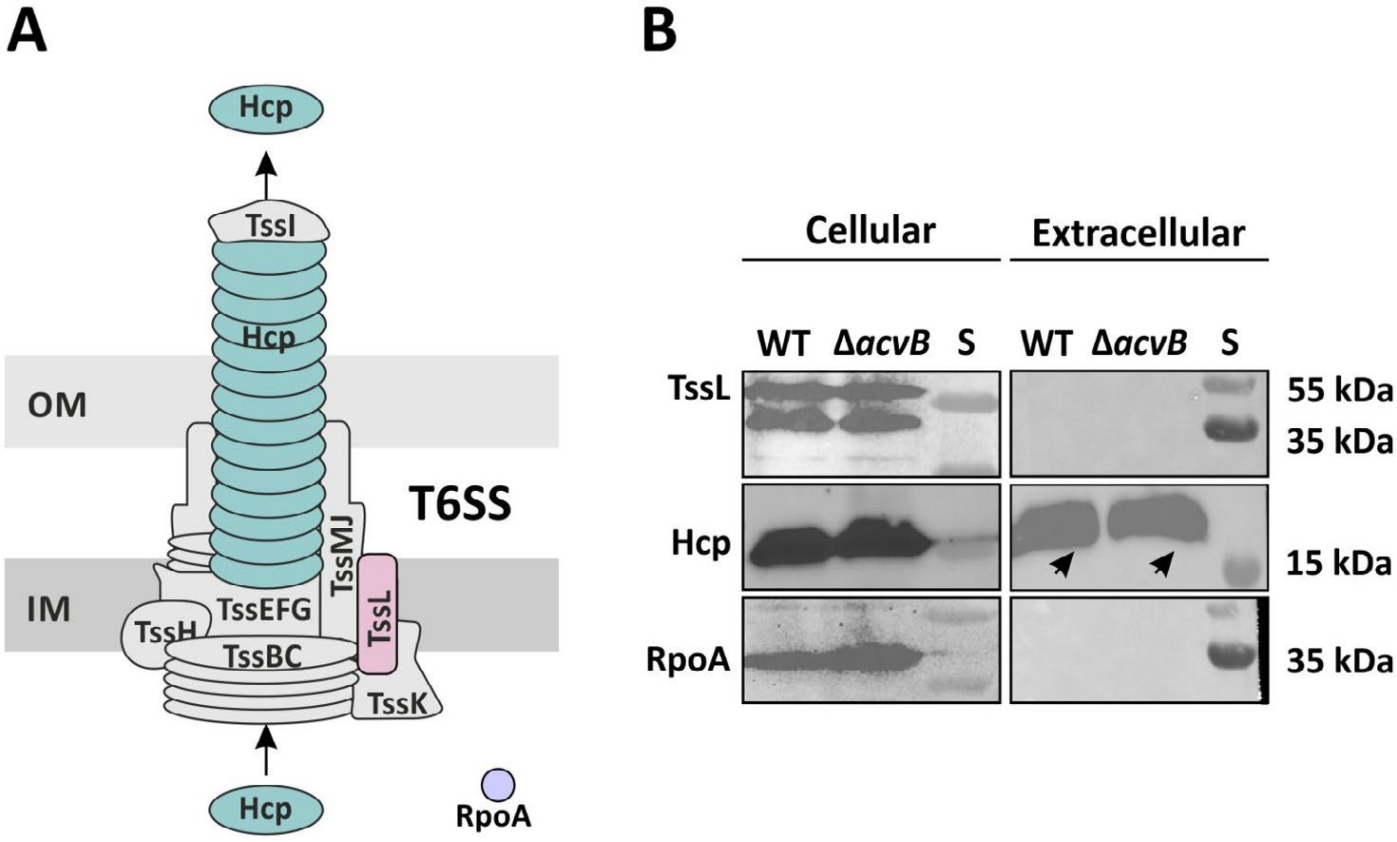
Evaluation of the presence and functionality of the type VI secretion system (T6SS) by Western blot analysis. (A) Schematic representation of *A. tumefaciens* T6SS, adapted from (Bernal et al. 2018). Components detected by Western blot are highlighted. (B) Western blotting was performed using antibodies against TssL, an inner membrane component of the T6SS, and Hcp, a structural protein that forms the tail tube and is secreted by the system. TssL served as a marker for the presence of the T6SS machinery, while Hcp indicated functional secretion activity. To induce T6SS activity, strains were grown under acidic conditions, and both cell extracts and supernatants were analyzed. RpoA, a non‐secreted cytoplasmic protein, was used as a loading control for cell extracts and to verify the absence of cellular contamination in the supernatant samples. S: Protein standard.

To assess whether increase in L‐PG levels specifically affect T‐DNA transfer or have a broader impact on transmembrane‐spanning secretion systems, we analyzed the effect of altered L‐PG/PG levels on the type VI secretion system (T6SS), which is primarily involved in interbacterial competition within the plant host in *A. tumefaciens* (Ma et al. 2014). In particular, we examined the presence and functionality of the T6SS in the C58 *acvB* deletion mutant. For this, we used antibodies against the inner membrane component TssL and Hcp, a tail tube component and a secreted marker of T6SS activity (Figure 7A). The strains were grown under acidic conditions to induce Hcp secretion by the T6SS, and cell extracts and supernatants were analyzed via Western blot (Figure 7). If the T6SS was functional, we expected to detect Hcp in the supernatant. Conversely, if the system was defective, Hcp would either be absent or less abundant in the supernatant. RpoA, a non‐secreted cytosolic protein, was used to verify equal loading of cell extracts and to confirm the absence of contamination in the supernatant.

As shown in Figure 7, the T6SS was present in both the wild‐type and the *acvB* mutant, as indicated by the detection of TssL and Hcp in the cell extracts. Furthermore, the system appears to be functional in the *acvB* mutant, as evidenced by similar levels of Hcp secretion into the supernatant compared to the wild‐type. These results suggest that the imbalance in L‐PG/PG levels does not affect the T6SS formation and functionality but rather has a specific impact on T4SS.

### 2.6 The RP4 Conjugative T4SS Remains Functional in the *acvB* Mutant

To address whether the L‐PG effect is specific to the T4SS T‐DNA delivery, we examined whether other T4SSs, such as the conjugative RP4 system, might also be impaired due to their structural and functional similarity. Supporting this idea, a central feature of T4SSs is their extracellular pilus. Both the VirB/VirD4 and conjugative pili utilize related pilin subunits and share conserved assembly mechanisms, including a 1:1 interface between pilin monomers and PG (Costa et al. 2016; Zheng et al. 2020; Amro et al. 2023; Kreida et al. 2023; Vadakkepat et al. 2024). Importantly, PG is not merely a membrane lipid but is directly integrated into the pilus structure, where it stabilizes pilin interactions. We therefore hypothesized that the conjugative T4SS, particularly its pilus, may, like the VirB/VirD4 system, be functionally compromised in the *acvB* mutant due to altered PG/L‐PG ratios.

**FIGURE 8:**
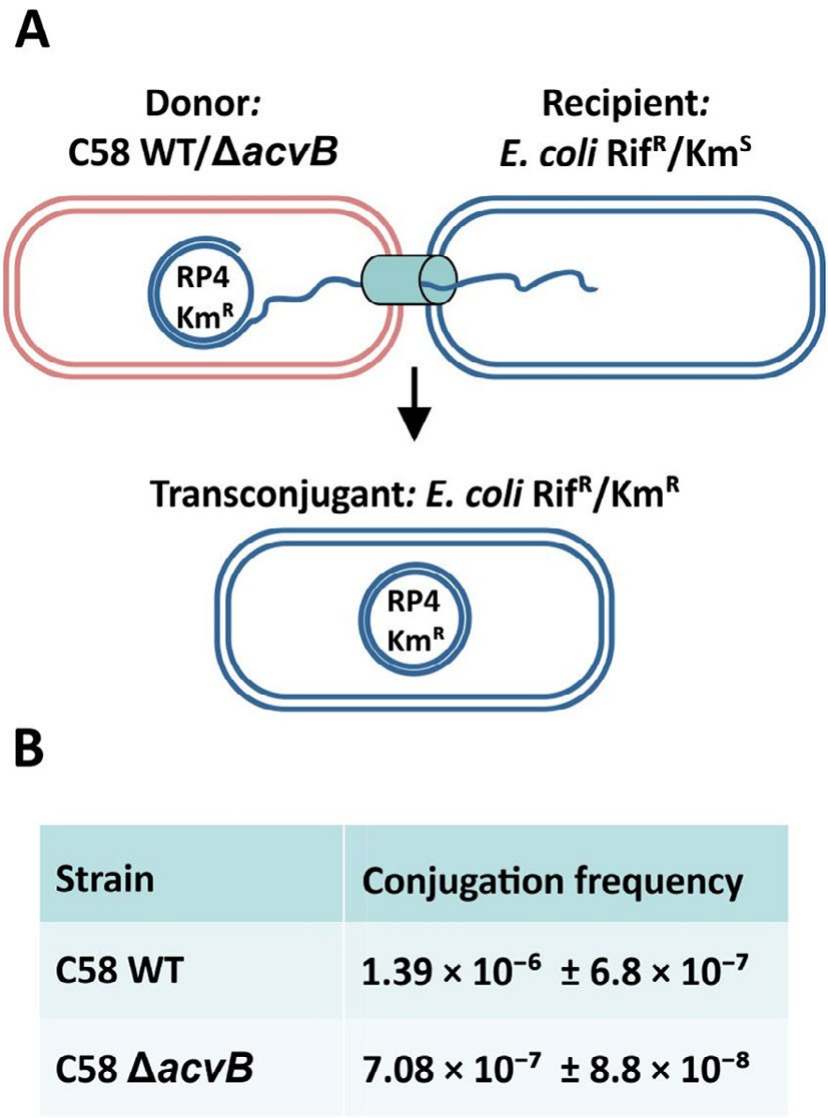
Conjugation efficiency of *A. tumefaciens* C58 wild‐type and Δ*acvB* mutant. (A) Schematic overview of the conjugation assay using the broad‐host‐range plasmid RP4, transferred from *A. tumefaciens* donors to rifampicin‐resistant (Rif^R^), kanamycin‐sensitive (Kan^S^) *E. coli* C600 recipients. (B) Conjugation frequencies (transconjugants per donor) of wild‐type and Δ*acvB* strains. Data represent mean ± SD from three independent experiments.

To test this hypothesis, we performed conjugation assays in *A. tumefaciens* C58 using the broad‐host‐range plasmid RP4, which encodes the P‐type pilus as well as all essential transfer genes and confers resistance to kanamycin, tetracycline, and ampicillin. RP4 was transferred from either wild‐type C58 or the *acvB* mutant to rifampicin‐resistant *E. coli* C600 (Figure 8A). Transconjugants were selected on LB agar plates containing both rifampicin and kanamycin, and conjugation frequencies were subsequently determined. To promote maximal L‐PG synthesis and thus enhance the lipid imbalance phenotype, all donor strains were grown in AB‐MES medium adjusted to pH 5.5 prior to the assay.

Both the wild‐type and *acvB*‐mutant strains successfully transferred the RP4 plasmid to *E. coli* at comparable efficiencies (Figure 8B). Although the mutant exhibited a modest (~2‐fold) reduction in average transfer frequency, the variability among individual replicates, with some matching or even exceeding wild‐type transfer rates, suggests that the altered L‐PG/PG membrane composition in the absence of *acvB* does not substantially impair the function of the RP4 T4SS.

## 3 Discussion

### 3.1 The L‐PG Homeostasis System LpiA/AcvB in *A. tumefaciens*

Membrane remodeling is a central adaptive strategy that enables bacteria to cope with fluctuating environmental conditions. In *A. tumefaciens* , the LpiA/AcvB system contributes to the homeostasis of L‐PG, particularly under acidic conditions when L‐PG levels increase (Groenewold et al. 2019). Since the *A. tumefaciens lpiA* mutant, which is unable to synthesize L‐PG, does not display a growth defect under acidic conditions (Groenewold et al. 2019), L‐PG synthesis appears to be nonessential for survival or growth at low pH. Instead, L‐PG accumulation likely represents a facultative or compensatory response that fine‐tunes membrane properties rather than providing primary protection.

In contrast, deletion of *acvB* causes strong growth impairment at low pH, coinciding with excessive L‐PG accumulation (Groenewold et al. 2019). Together, these observations highlight that the regulation of L‐PG levels, rather than its synthesis per se, is critical for maintaining membrane integrity under acid stress. AcvB plays a central role in this process by catalyzing the hydrolysis of L‐PG back to PG, thereby maintaining the optimal L‐PG/PG ratio and ensuring proper membrane charge. This reaction also releases free lysine into the periplasm, which may act as a local proton buffer and contribute to pH stabilization.

Disruption of this balance through the loss of AcvB likely results in membrane disorganization and impaired function of membrane‐associated processes. Hence, AcvB serves as a key regulator that prevents deleterious L‐PG accumulation, ensuring membrane stability and proper physiological adaptation under acidic conditions.

In a recent study, the structure of the mature *A. tumefaciens* AcvB has been investigated, revealing important details about its interaction with LpiA and the membrane (Hoshi and Watanabe 2025). Both the N‐terminal and C‐terminal domains adopt an α/β hydrolase fold. The N‐terminal domain forms a six‐stranded parallel β‐sheet surrounded by six α‐helices, while the C‐terminal domain forms a seven‐stranded parallel β‐sheet surrounded by eight α‐helices (Hoshi and Watanabe 2025). The authors show that the C‐terminal domain interacts with LpiA and with the membrane, whereas the N‐terminal domain does not, leaving the function of the N‐terminal domain still elusive. The interaction between the C‐terminal domain and LpiA orients the AcvB C‐terminal domain such that its protruding loop, containing two essential hydrophobic residues, associates with the membrane and recognizes the acyl chains of L‐PG. Thus, AcvB and LpiA function cooperatively in regulating Lys‐PG metabolism.

### 3.2 VirJ and AcvB: L‐PG Hydrolases Supporting T4SS‐Mediated T‐DNA Transfer

The AcvB homolog VirJ is a Ti plasmid‐encoded virulence factor in *A. tumefaciens* that has long been associated with T‐DNA transfer, although its precise molecular function remained elusive. Its homology to AcvB suggests that VirJ may perform a similar role in maintaining the L‐PG balance in the membrane. This balance is not only critical for adaptation to acid stress but also for the proper function of the T4SS (Groenewold et al. 2019), which depends on a stable membrane environment for its activity.

In this study, we show that VirJ also possesses L‐PG hydrolase activity, uncovering a conserved mechanism by which both VirJ and AcvB modulate the membrane phospholipid composition to facilitate efficient T4SS‐mediated T‐DNA transfer. Our previous work demonstrated that the N‐terminal domain of AcvB is dispensable for T‐DNA transfer and tumorigenesis. Deletion of this region did not affect enzymatic activity or virulence, indicating that the C‐terminal catalytic domain is sufficient to support T4SS function (Groenewold et al. 2019). Our finding that VirJ fully complements AcvB loss by lowering elevated L‐PG levels, rescuing acid‐sensitive growth defect and restoring T‐DNA transfer, strongly supports L‐PG hydrolysis as essential for T4SS‐mediated DNA delivery. While the N‐terminal domain of AcvB may play auxiliary roles, such as modulating protein localization or stability, it is not essential under the tested conditions (Groenewold et al. 2019). Future studies might explore whether this domain becomes functionally relevant under specific environmental or host‐associated stresses.

In this context, it is noteworthy that, similar to *A. tumefaciens* AcvB, the homologous *Brucella abortus* protein VirJ also adopts a two‐domain architecture and contributes to T4SS function. VirJ is essential for the efficient secretion of at least two VirB substrates, SepA and Bpe123, which transit through a periplasmic intermediate. Although VirJ is not required for their periplasmic localization, it is crucial for secretion across the outer membrane. These observations suggest the existence of a secretion complex in which VirJ functions as a central organizational component (del Giudice et al. 2016).

The structure of the N‐terminal domain of *Brucella* VirJ has also recently been solved (Dugelay et al. 2025). Interestingly, this domain lacks the catalytic triad characteristic of active hydrolases, which is present in the C‐terminal domain of *Agrobacterium* AcvB and in single‐domain VirJ proteins. Its retention in two‐domain AcvB proteins may reflect an evolutionary remnant of an ancestral hydrolase that acted on other amino‐acylated lipids. Although catalytically inactive, the N‐terminal domain could contribute to structural stability or facilitate interactions with other proteins or substrates. Notably, it is dispensable for both L‐PG hydrolase activity and T‐DNA transfer by the T4SS in *A. tumefaciens* , suggesting that its role is primarily structural or modulatory rather than directly catalytic.

### 3.3 L‐PG Hydrolase Activity of VirJ or AcvB Is Required for T‐DNA Transfer by the VirB/VirD4 T4SS but Dispensable for the Function of the RP4 T4SS and T6SS

Although lipid modifications broadly impact the functionality of membrane integrated machineries, our results show a remarkable specificity, with T4SS function strictly depending on L‐PG hydrolysis, while RP4 T4SS and T6SS remain unaffected.

Membrane lipid modifications, such as PG aminoacylation, can broadly influence the assembly and activity of membrane‐associated systems. Aminoacylation of the anionic phospholipid PG yields zwitterionic or cationic derivatives, such as alanyl‐PG or L‐PG, which reduce membrane surface charge and modulate interactions with cationic antimicrobial peptides (CAMPs) and other positively charged compounds (den Kamp et al. 1969; Haest et al. 1972; Roy 2009). Disruption of this balance, as shown in *P. aeruginosa* , can sensitize cells to antibiotics, highlighting the need for tight regulation of lipid composition to maintain membrane integrity (Hebecker et al. 2011; Arendt et al. 2012). Moreover, lipid imbalances by changing the head group or fatty acid tail composition impact a wide range of membrane‐associated processes, including secretion, transport, stress responses, and signal transduction (Wessel et al. 2006; Klüsener et al. 2009; Aktas et al. 2014; Koch et al. 2019; Kamel et al. 2022; Lee et al. 2024).

Highlighting the importance of head group identity, another unusual phospholipid, phosphatidylcholine (PC), in *A. tumefaciens* also modulates the T4SS. PC is a fully head group methylated derivative of phosphatidylethanolamine (Sohlenkamp et al. 2003). Loss of PC impairs the production of T4SS components, likely due to dysfunction of the VirA/VirG two‐component system and subsequent failure of *vir* gene expression (Wessel et al. 2006; Klüsener et al. 2009; Aktas et al. 2014). In contrast, increased L‐PG levels in the *acvB* mutant do not prevent synthesis and assembly of T4SS, as key VirB components and the extracellular T‐pilus are detected by Western blot analysis. This indicates that the L‐PG hydrolase activity of AcvB/VirJ is not required for T4SS biogenesis but instead affects T4SS activity at a post‐assembly level. Nevertheless, the system remains nonfunctional, and it is unresolved whether the detected components assemble into a fully functional secretion complex (Groenewold et al. 2019). These findings emphasize that membrane lipids differentially affect both the formation and function of the T4SS.

The insensitivity of the T6SS to altered L‐PG levels likely reflects fundamental architectural and functional differences. Unlike T4SSs, the T6SS does not assemble an extracellular pilus. Instead, it forms a contractile nanomachine analogous to phage tails, with a cytoplasmic baseplate and sheath that propels an inner tube across the envelope into target cells (Ma et al. 2009; Ho et al. 2014; Zoued et al. 2014; Wu et al. 2020; He et al. 2023). As no direct stoichiometric lipid‐protein interactions have been described for T6SS components, its function appears independent of the precise membrane lipid environment.

Given these differences, a key question arises: what makes the VirB/VirD4 system uniquely vulnerable to L‐PG accumulation? Recent cryo‐EM studies have revealed that the *A. tumefaciens* VirB/VirD4 T4SS pili incorporate an equimolar ratio of the VirB2 pilin and PG, where PG molecules stabilize the pilus architecture through electrostatic interactions with VirB2 (Kreida et al. 2023). Although similar PG‐pilin interactions have been shown to be important for pilin stability in conjugative T4SSs (Zheng et al. 2020; Costa et al. 2024; Vadakkepat et al. 2024), our findings demonstrate that the RP4‐encoded conjugation system remains functional in *acvB* mutants with elevated L‐PG levels. This unexpected resilience suggests that, despite their structural and mechanistic homology, fundamental differences exist between virulence‐associated and conjugative T4SSs. Importantly, although the extracellular T‐pilus is produced in the *acvB* mutant, the altered PG:L‐PG ratio in the membrane may compromise its structural integrity or function, indicating that pilus formation does not necessarily reflect a fully operational T4SS. However, several mechanistic scenarios may explain the unique vulnerability of the VirB/VirD4 system: (i) Displacement of PG from VirB2: L‐PG, a cationic derivative of PG, may compete for PG binding during pilus assembly but fail to support proper pilus structure/function due to its bulkier, charged head group. (ii) Electrostatic interference: L‐PG accumulation alters membrane surface charge, potentially disrupting electrostatic interactions essential for the activity of inner membrane components such as VirB4 or VirB11. (iii) Disruption of membrane microdomains: The VirB/VirD4 system localizes to detergent‐resistant membrane microdomains enriched in specific lipids (Czolkoss et al. 2021). L‐PG may disrupt these microdomains by altering membrane curvature, fluidity, or lipid‐protein interactions.

In contrast, the conjugation system may benefit from the general robustness of F‐pili, which have evolved to function under harsh environmental conditions, including mechanical and thermal stress (Patkowski et al. 2023). Alternatively, localized pools of PG at the conjugation site may buffer against global lipid perturbations. These differences likely reflect evolutionary adaptations. Virulence‐associated T4SSs may be finely tuned to plant host conditions, requiring precise lipid‐protein interactions, whereas conjugative systems are optimized for broad host range and membrane variability. Additionally, a comparative analysis of T‐, N‐, and F‐pili revealed that, despite their structural similarities, they differ in charge, phospholipid composition, width, and lumen, supporting the hypothesis that these pili can be differentially influenced by membrane lipid imbalances (Amro et al. 2023; Costa et al. 2024). Moreover, even though general features of the RP4 T4SS and T‐type pili are similar, they serve different functions. The T‐type pilus mediates the transfer of the T‐DNA complex into plant cells, whereas the RP4 pilus primarily facilitates cell–cell interactions. However, recent studies have shown that plasmid DNA can also be transferred via conjugation pili between physically distant bacterial cells (Goldlust et al. 2023).

Interestingly, in *Bacillus subtilis* , a Gram‐positive bacterium with naturally high L‐PG levels, elevated L‐PG enhances conjugation, while its reduction impairs transfer (Johnson and Grossman 2016). In contrast, in the Gram‐negative *A. tumefaciens* with low basal L‐PG, the RP4 conjugation system remains functional under both reduced and elevated L‐PG levels. These distinctions likely arise from differences in conjugation mechanisms. Gram‐positive systems often involve direct membrane contacts sensitive to lipid composition, whereas Gram‐negative systems like RP4 utilize pilus‐mediated DNA transfer that appears more resilient to lipid alterations. Thus, membrane lipid composition plays a more limited role in conjugation efficiency in Gram‐negative bacteria than in their Gram‐positive counterparts.

### 3.4 Why Keep Two L‐PG Hydrolases? VirJ as a Virulence‐Optimized Derivative of AcvB?

Why do octopine‐type *A. tumefaciens* strains maintain two L‐PG hydrolases, AcvB and VirJ, while VirJ is absent in nopaline‐type strains? This raises key questions about potential specialization, regulatory divergence, or functional complementarity.

The chromosomal *acvB* gene is conserved across all *A. tumefaciens* strains, regardless of Ti plasmid type (Weisberg et al. 2023). It encodes a two‐domain protein comprising a C‐terminal L‐PG hydrolase domain essential for membrane lipid homeostasis and an N‐terminal domain of unknown function (Groenewold et al. 2019). In contrast, *virJ*, located exclusively on octopine‐type Ti plasmids, encodes a truncated variant of AcvB that includes only the catalytic domain. These structural and genomic differences suggest distinct regulatory and functional roles. Under host‐inducing conditions, *virJ* expression is upregulated via the VirA/VirG system, enabling temporary enhancement of membrane remodeling during T‐DNA transfer. Meanwhile, constitutive expression of *acvB* ensures continuous membrane lipid balance, supporting general cellular viability and T4SS function.

The coexistence of AcvB and VirJ in octopine‐type strains suggests a context‐dependent specialization. AcvB maintains lipid homeostasis under standard conditions, especially at low pH, while VirJ provides an infection‐specific boost to L‐PG hydrolysis, likely enabling rapid membrane adaptation for efficient T4SS assembly and host colonization. The restriction of *virJ* to octopine‐type Ti plasmids indicates a selective advantage under particular host or environmental conditions. Its origin, probably from a truncated *acvB* (Weisberg et al. 2023), reflects evolutionary pressure to optimize membrane remodeling during infection, possibly related to host specificity, wound microenvironment adaptation, or enhanced plasmid transmission.

Overall, VirJ and AcvB represent an adaptive evolutionary strategy in *A. tumefaciens* to balance membrane lipid homeostasis with the specific demands of host interaction and horizontal gene transfer. By modulating L‐PG levels through hydrolase activity, these proteins ensure proper function of the T4SS, highlighting the importance of lipid remodeling in bacterial virulence.

## 4 Material and Methods

### 4.1 Plasmid, Strains and Media

The plasmids and strains used in this study are listed in Table S1. *Escherichia coli* strains were cultured at 37°C in LB medium (Sambrook and Russell 2006), with ampicillin (100 μg mL^−1^), streptomycin (100 μg mL^−1^), or spectinomycin (300 μg mL^−1^) added as needed. *Agrobacterium tumefaciens* strains were grown at 30°C in either YEB‐complex or AB‐MES minimal medium (pH 5.5) (Schmidt‐Eisenlohr et al. 1999). For pre‐cultures, *A. tumefaciens* strains were incubated overnight at 30°C in YEB liquid medium, supplemented with 300 μg mL^−1^ spectinomycin if required. The pre‐cultures were then transferred to AB‐MES minimal medium to establish the main cultures.

### 4.2 Plasmid Construction

For VirJ overproduction and purification, the expression vector pET22b(+)Strep (Nicke et al. 2013) was employed. This vector enables the fusion of an N‐terminal PelB signal sequence for targeting the protein to the periplasm and a C‐terminal Strep‐tag II for purification via affinity chromatography. The *virJ* gene fragment, encoding amino acids 23‐233, was PCR‐amplified from a synthetic gene fragment (Life Technologies GmbH, Carlsbad, CA, USA) using oligonucleotides 1 and 2 (Table S1). The resulting PCR product was inserted into the NcoI/HindIII restriction sites of pET22b(+)Strep, generating the expression vector pET22b(+)Strep_pelB_virJΔaa1‐22.

To investigate the subcellular localization of VirJ, we constructed the vector pTrc200_virJ‐phoA. This vector contains a DNA fragment with the predicted signal sequence of *virJ*, as identified by SignalP6.0 (Teufel et al. 2022), fused to a *phoA* gene that lacks its intrinsic signal sequence. The corresponding DNA fragment was synthesized by Life Technologies GmbH (Carlsbad, CA, USA) (Table S1). The In‐Fusion HD cloning method was employed to insert this sequence into the plasmid pTrc200. Corresponding vectors, lacking a secretion signal or carrying the native PhoA secretion signal, were used as controls (Groenewold et al. 2019).

The InFusion HD cloning method with primers 3 and 4 (Table S1) was used to clone the full‐length *virJ* gene into the shuttle vector pTrc200 yielding plasmid pTrc200_VirJ. Mutagenized plasmids pTrc200_virJ_S60A, pTrc200_virJ_S127A, and pTrc200_virJ_H223N were constructed by site‐directed mutagenesis (primers 5–7, Table S1) using the QuikChange kit (Agilent) according to the manufacturer's instructions.

Sequencing was used to check the insert after mutagenesis and cloning. All sequence determinations were performed by the external service provider Microsynth SEQLAB (Göttingen, Germany) via Sanger sequencing.

### 4.3 Analysis of the Growth Behavior of Different *A. tumefaciens* Strains Under Acidic Condition

The growth of different *A. tumefaciens* strains was monitored in AB‐MES medium (pH 5.5) at 30°C. Pre‐cultures were prepared in YEB medium, and for pTrc_VirJ‐containing strains, 300 μg mL^−1^ spectinomycin and 100 μg mL^−1^ streptomycin were added. Main cultures were started at an initial OD_578_ of 0.2 without antibiotics, with 0.4 mM IPTG added to induce pTrc_VirJ in the complemented Δ*acvB* strains. Growth was tracked by measuring OD_578_ at 1.5, 3, 4, 5, 6, and 24 h. After 24 h, cultures were adjusted to an OD_578_ of 3.0, and 2 mL samples were harvested for subsequent two‐dimensional thin‐layer chromatography (2D‐TLC) analysis.

### 4.4 Lipid Extraction and 2D‐TLC Analyses

Phospholipids from different *A. tumefaciens* strains grown in AB‐MES medium (pH 5.5) were extracted following the method of Bligh and Deyer (1959). In brief, 2 mL cultures with an OD_578_ adjusted to 3.0 were collected by centrifugation and washed with 500 μL of water. The cells were resuspended in 100 μL of water and homogenized with 375 μL of methanol/chloroform (2:1). Subsequently, 100 μL of water and 100 μL of chloroform were added, and the mixture was vortexed briefly before centrifugation at 15.700 × g for 5 min. The lower organic phase was recovered, dried under vacuum, and the resulting lipid pellets were resuspended in 15 μL of methanol/chloroform (1:1). The lipid extracts were then spotted onto an HPTLC silica gel 60 plate (Merck, Darmstadt, Germany) and analyzed by 2‐D TLC using two different solvent systems: (1) the first dimension consisted of methanol/chloroform/water (30:12.5:2, v/v/v), while the second dimension used chloroform/methanol/acetic acid/water (40:6:7.5:2, v/v/v/v), or (2) the first dimension used a solvent mixture of chloroform/methanol/water (65:25:4, v/v/v), and the second dimension used chloroform/methanol/acetic acid/water (90:15:10:3.5, v/v/v). Phospholipids were visualized using 5% (w/v) molybdatophosphoric acid in ethanol, and lysine‐containing phospholipids were detected using 2% (w/v) ninhydrin in ethanol.

### 4.5 *Agrobacterium*‐Mediated Transient Transformation in *Arabidopsis* Seedlings

The T‐DNA transfer capability of the different *A. tumefaciens* strains into plant cells was assessed using *Arabidopsis* seedling infection assays based on the AGROBEST method (Wu, Liu, et al. 2014). The T‐DNA vector pBISN1, which contains the *gusA*‐intron (Narasimhulu et al. 1996), was introduced into different *A. tumefaciens* C58 strains (wild‐type, Δ*acvB*, and Δ*acvB* complemented with pTrc‐VirJ derivatives) via electroporation. These strains were then used to infect 7‐day‐old *Arabidopsis* seedlings. After 3 days of infection, the efficiency of transient GUS expression was assessed through GUS staining. A detailed description of the assay is provided in (Wu, Liu, et al. 2014; Groenewold et al. 2019). For lipid analysis of the strains used for the infection assays, 2 mL samples with an OD_578_ adjusted to 3.0 were utilized.

### 4.6 Time Course Western Blot Analysis of VirB Proteins in *A. tumefaciens* Cell Extracts

Precultures of *A. tumefaciens* strains grown in LB complex medium were diluted to an OD_578_ of 0.1 in AB‐MES minimal medium (pH 5.5) supplemented with 80 mM MgCl_2_ (required for growth of the Δ*acvB* mutant at pH 5.5) and incubated overnight. Main cultures were then prepared from these precultures in AB‐MES minimal medium (pH 5.5) containing 80 mM MgCl_2_ and 200 μg mL^−1^ acetosyringone and incubated at 23°C. Samples were harvested 4, 8, and 16 h after acetosyringone addition. Cell densities were determined by measuring OD_578_, and samples were normalized to equivalent optical densities prior to processing. Normalized samples were processed for SDS‐PAGE using standard procedures (12.5% SDS‐polyacrylamide gels), followed by Western blot analysis. VirB proteins were detected using *A. tumefaciens* ‐specific antisera against VirB2, VirB5, VirB8, and VirB9 (1:10,000 dilution). RpoA was detected as an internal loading control using anti‐RpoA antiserum (1:9500). Detection was performed using a goat anti‐rabbit IgG HRP‐conjugated secondary antibody (Bio‐Rad, Hercules, CA, USA).

### 4.7 Detection of the Extracellular T‐Pilus Component VirB2

The assay was performed based on (Wu, Chen, and Lai 2014), with some modifications. Briefly, *A. tumefaciens* cells from overnight LB cultures were harvested and resuspended in liquid AB‐MES minimal medium (pH 5.5) supplemented with 80 mM MgCl_2_ (required for growth of the Δ*acvB* mutant at pH 5.5), followed by incubation for 4 h. Cell densities were then adjusted, and 200 μL of the bacterial suspension was spread onto solid AB‐MES medium (pH 5.5) containing 80 mM MgCl_2_ and 200 μg mL^−1^ acetosyringone for *vir* gene induction. Plates were incubated at 19°C for 3 days. For sample preparation, bacterial cells were scraped from the AB‐MES agar plates and resuspended in 2 mL of 10 mM phosphate buffer, pH 5.3. The OD_578_ was adjusted to 6, and cell suspensions were either left unsheared or sheared by passage through a 20‐gauge needle syringe five times. Samples were then centrifuged at 15,700 × g for 10 min at 4°C to collect the supernatant enriched in T‐pilus material, designated as the extracellular fraction (EC). Supernatants were filtered through a 0.22‐μm sterile filter to remove residual bacterial cells and precipitated overnight with 150 μL trichloroacetic acid (100%, w/v) and 30 μL sodium deoxycholate (1%, w/v). Precipitated samples were resuspended in 500 μL SDS (2%, w/v) and 150 μL acetone (100%) and incubated at −20°C for 30 min, followed by centrifugation (30 min at 15,700 × g). The resulting pellets were washed with 100% acetone before they were dried in a SpeedVac concentrator. The dried samples were resuspended in 25 μL 5× SDS sample buffer. The OD normalized sheared cell pellets (designated as the intracellular fraction, IC) were resuspended in 500 μL 1× SDS sample buffer. Aliquots of IC and EC samples (5 μL) were separated by standard SDS‐PAGE (12.5% SDS gel) and Western blotting as described above.

### 4.8 Analysis of T6SS Functionality via Cellular and Secreted Protein Profiling in *A. tumefaciens* WT and *acvB* Mutant

To assess T6SS functionality in *A. tumefaciens* through cellular and secreted protein profiling, we followed the method described by (Wu et al. 2008), with some modifications. The wild‐type and *acvB* mutant strains of *A. tumefaciens* were cultured overnight at 30°C in AB‐MES medium (pH 5.5) supplemented with 80 mM MgCl_2_. The addition of MgCl_2_ was used to compensate for the reduced growth efficiency of the *acvB* mutant under acidic conditions, as previously described (Groenewold et al. 2019). The next day, all cultures were adjusted to the same OD at 578 nm. For the preparation of cellular protein samples, 1 mL of each culture was harvested by centrifugation at 15,700 × g for 10 min at 4°C. The resulting cell pellets were washed once with sterile water and subsequently resuspended in 100 μL of 1× SDS sample buffer. For the preparation of secreted protein samples, 2 mL of each OD‐normalized culture was centrifuged at 15,700 × g for 10 min at 4°C. From each sample, 1.8 mL of the supernatant was carefully collected and sterile‐filtered using a 0.22 μm membrane filter. To 1.5 mL of the filtered supernatant, 45 μL of 1% (w/v) sodium deoxycholate (DOC; Thermo Scientific) was added and mixed thoroughly by vortexing. Samples were incubated on ice for 10 min, followed by the addition of 225 μL cold 100% (w/v) trichloroacetic acid. After vortexing, samples were incubated overnight on ice. The following morning, samples were centrifuged at 15,700 × g for 60 min at 4°C. Supernatants were carefully removed, and the resulting protein pellets were dried in a SpeedVac concentrator for 1.5 h. Pellets were then centrifuged again for 15 min at 15,700 × g, any remaining liquid was removed, and the pellets were dried for an additional hour. Dried protein pellets were resuspended in 10 μL of 1 M Tris base (original pH), and 10 μL of 5 × SDS sample buffer was added to each sample. Subsequently, 15 μL of each cellular and supernatant sample was subjected to SDS‐PAGE, followed by Western blot analysis using standard protocols. Primary antibodies were used against RpoA (1:9500), Tssl (1:1000), and Hcp (1:2500). A goat anti‐rabbit IgG secondary antibody was applied as described above.

### 4.9 Conjugative Transfer of the RP4 Plasmid From *A. tumefaciens* to *E. coli* C600

Because L‐PG synthesis in *A. tumefaciens* is promoted under acidic conditions, conjugation assays were conducted in liquid AB‐MES medium (pH 5.5) with early stationary‐phase cultures of *A. tumefaciens* WT/RP4 or *acvB*/RP4 (Kan^R^, Tet^R^, Amp^R^; donors) and rifampicin‐resistant *E. coli* C600 (Rif^R^; recipient). Growth of the *acvB* mutant at low pH was supported by adding MgCl_2_ (80 mM), which rescues this defect via divalent cation supplementation (Groenewold et al. 2019). MgCl_2_ was included in both wild‐type and mutant cultures.

For conjugation, 4 × 10^8^ donor cells were mixed with 5.6 × 10^8^ recipient cells and incubated at 30°C for 4 h. The reaction was stopped by vortexing the mixture for 1 min. Serial dilutions were plated onto LB agar containing rifampicin (100 μg mL^−1^) and kanamycin (50 μg mL^−1^) to select for transconjugants. Plates were incubated overnight at 37°C, and colony formation was assessed the following day. Conjugation efficiency was determined by quantifying the number of transconjugant colonies.

### 4.10 Subcellular Localization of VirJ in *A. tumefaciens* C58

To examine the localization of VirJ in *A. tumefaciens* C58, we constructed a plasmid (pTrc200_virJ‐phoA) encoding a translational fusion of the predicted N‐terminal signal sequence of VirJ with PhoA lacking its native signal peptide. PhoA becomes enzymatically active only after being translocated to the periplasm. Control plasmids, pTrc200_native‐phoA and pTrc200_Δss‐phoA (Groenewold et al. 2019), were included in the analysis. These constructs were used to assess periplasmic alkaline phosphatase activity by monitoring the hydrolysis of 5‐bromo‐4‐chloro‐3‐indolyl phosphate (BCIP) in the *A. tumefaciens* C58 wild‐type strain. The hydrolysis product, 5‐bromo‐4‐chloro‐3‐indoxyl, is oxidized to produce the blue dye 5,5′‐dibromo‐4,4′‐dichloro‐indigo (Brickman and Beckwith 1975). The assay was performed on YEB plates containing 90 μg mL^−1^ BCIP and 25 mM disodium hydrogen phosphate to inhibit the activity of the endogenous alkaline phosphatase in *A. tumefaciens* (Bina et al. 1997). The plates were inoculated with overnight cultures of the appropriate *A. tumefaciens* strains and incubated at 30°C for 4 days.

### 4.11 Production and Purification of Recombinant VirJ From the Periplasmic Fraction of *E. coli* BL21

*E. coli* BL21 harboring the plasmid pET22b(+)Strep_pelB_virJ Δaa1‐22 was cultured in 500 mL of LB medium supplemented with 100 μg mL^−1^ ampicillin. The culture was grown to an OD_578_ of 0.5, at which point protein expression was induced with 25 μM IPTG and allowed to proceed for 24 h at 17°C. After induction, cells were harvested, resuspended in 50 mM HEPES‐NaOH (pH 8.0), 150 mM NaCl, and 20% (w/v) D‐(+)‐sucrose, and the pellets were stored at −20°C. For protein extraction, the thawed cells were resuspended in the same buffer supplemented with 2 mg mL^−1^ polymyxin B (Sigma‐Aldrich) and incubated at 4°C for 1.5 h. The periplasmic fraction was then collected by centrifugation at 20,000 × g for 1.5 h at 4°C. To mask biotinylated host proteins in the supernatant, 0.4 μM avidin (Calbiochem, Merck) was added. The soluble periplasmic fraction was subsequently applied to 1 mL of Strep‐Tactin Superflow resin (IBA), pre‐equilibrated with washing buffer (50 mM HEPES‐NaOH, pH 8.0, 100 mM NaCl). The resin was washed with 2 × 5 mL of the same buffer, and the target protein was eluted using 2.5 mM desthiobiotin (IBA). Elution fractions were dialyzed against 20 mM HEPES‐NaOH (pH 6.8) and concentrated using Vivaspin 4 concentrators (10‐kDa cut‐off, Sartorius), and protein concentrations were determined using the Bradford reagent (Sigma‐Aldrich).

### 4.12 VirJ In Vitro Activity Assay

To evaluate the enzymatic activity of VirJ, an in vitro L‐PG hydrolysis assay was performed. A micellar substrate solution containing L‐PG was prepared by mixing 2.5 mg of 18:1 L‐PG (1,2‐dioleoyl‐*sn*‐glycero‐3‐[phospho‐*rac*‐(3‐lysyl(1‐glycerol))]), Avanti Polar Lipids with 1 mL of dialysis buffer containing 33 mg Triton X‐100 (Sigma‐Aldrich), then incubating at 37°C for 15 min under vigorous agitation. The standard assay of 40 μL comprised 20 μL of 1 μM of VirJ solution in 20 mM HEPES‐NaOH (pH 6.8) and 20 μL of the L‐PG micellar substrate solution. Reactions were incubated at 37°C with shaking at 1000 rpm. At intervals of 0, 0.5, 1, 2, 5, 10, 15, and 20 min, 5 μL samples were taken and the reaction mixtures were analyzed by one‐dimensional TLC according to (Arendt et al. 2013). L‐PG and L‐lysine were visualized by ninhydrin staining, with an L‐lysine standard included for comparison. Assays were performed in triplicate and yielded reproducible results. Enzymatically released lysine was quantified by densitometry.

## Author Contributions

M.A., J.M., F.N., D.J. conceptualized and supervised the experiments. D.J. and F.N. provided infrastructure. B.L., C.F., L.B., M.K.T., S.H. and J.B. conducted experiments and analyzed the data. M.A. and J.M. wrote the paper. All authors analyzed the results and approved the final version of the manuscript.

## Funding

This work was supported by Deutsche Forschungsgemeinschaft, MO 1749/1‐2, Research. Training Group 2341 “Microbial Substrate Conversion”.

## Ethics Statement

This study did not involve any experiments on animals or humans.

## Conflicts of Interest

The authors declare no conflicts of interest.

## Supporting information


**Figure S1:** Periplasmic localization of VirJ in *A. tumefaciens* .
**Figure S2:** Relative phospholipid levels in different *A. tumefaciens* strains.
**Figure S3:** Agrobacterium‐mediated transient transformation of *Arabidopsis* seedlings.
**Table S1:** Oligonucleotides and synthetic gene sequences.
**Table S2:** Strains and plasmids.

## Data Availability

All data are available in the main text and Supporting Information—S1.
