# Supplementary material for: The Ti Plasmid‐Encoded VirJ Functions as a Lysyl‐Phosphatidylglycerol Hydrolase in Agrobacterium tumefaciens
Source: Mol Microbiol. 2026 Mar 16;125(5):426–41. doi: 10.1111/mmi.70061 (PMC13135905; doi:10.1111/mmi.70061)
Supplement: Supplementary file 1 — Figure S1: Periplasmic localization of VirJ in A. tumefaciens . Figure S2: Relative phospholipid levels in different A. tumefaciens strains. Figure S3: Agrobacterium‐mediated transient transformation of Arabidopsis seedlings. Table S1: Oligonucleotides and synthetic gene sequences. Table S2: Strains and plasmids. [file MMI-125-426-s001.pdf]

Supplementary Information

The Ti plasmid-encoded VirJ functions as a lysyl-phosphatidylglycerol hydrolase in *Agrobacterium tumefaciens*

Britta Lotz[1], Lina Brodskaia[2], Christiane Fritz[2], Stefanie Hebecker[1], Maike K. Taucher[1], Jennifer Breitsch[1], Dieter Jahn[1], Franz Narberhaus[2], Jürgen Moser[1a] and Meriyem Aktas[2a]

[1]Institute for Microbiology, Technische Universität Braunschweig, 38106 Braunschweig, Germany

[2]Microbial Biology, Ruhr-Universität Bochum, 44780 Bochum, Germany

[a]M. A. and J. M. should be considered as joined senior author.

Correspondence: Meriyem Aktas (meriyem.aktas@rub.de)

Contents:

Supplementary Tables S1-S2

Supplementary Figures S1-S3

References to the supplement

Supplementary Tables

Table S1. Oligonucleotides and synthetic gene sequences

No.	Sequence (5’-3’)	Application
1	ccggcgatggccatggCAAATGATCGTGCAAATGGTGTTATGTGG	Construction of pET22b(+)Strep_pelB_VirJΔaa1-22
2	aagcgctgagaagcttCAGCGGAGCCGGACCGC	Construction of pET22b(+)Strep_pelB_VirJΔaa1-22
3	AGGAAACAGACC*ATGGCCATTAAACTGGTTCTGATCCTGGTTT TTACCCTGTTTCTGGCAGCAGATGCAGCCTATGCA*AATGATCG TGCAAATGGTGTTATGTGG (leader sequence in italic)	Construction of pTrc200_VirJ.
4	CTAGAGGATCCCCGGGTTATTTTTCGAACTGCGGGTGGCTC	Construction of pTrc200_VirJ.
5	CGTGGCCATTATCTATgccGGTGATGCAGGTTG	Construction of pTrc200_VirJS60A
6	GCTGATCGGTTATgccTTTGGTGCAGATGTTATGCCTGCC	Construction of pTrc200_VirJS127A
7	CGGTGGTCATgctTTTGGCAACG	Construction of pTrc200_VirJH223N
Sequence of synthetic *virJ* gene	AATGATCGTGCAAATGGTGTTATGTGGTCAAATGGTGGTGAAGCCGGTGTTCGTCTGCCG<br>CTGCGTGTTTTTAATGCAAAACCGGCAAAAAATACCGTGGCCATTATCTATAGCGGTGATG<br>CAGGTTGGCAGAATATTGATGAAGTTATTGGCACCTATCTGCAGACCGAAGGTATTCCGG<br>TTATTGGTGTTAGCAGCCTGCGTTATTTTTGGAGCGAACGTAGCCCGAGCGAAACCGCAA<br>AAGATCTGGGTCATATTATTGACGTGTACACCAAACATTTTGGCGTTCAGAATGTTCTGCT<br>GATCGGTTATAGCTTTGGTGCAGATGTTATGCCTGCCAGCTTTAATCGTCTGACCCTGGAA<br>CAGAAAAATCGCGTTAAACAAATTAGCCTGCTGGCACTGAGCCATCAGGTTGATTATGTTG<br>TTAGCTTTCGTGGTTGGCTGCAGCTGGAAACCGAAGGCAAAGGTGGTAATCCGCTGGAT<br>GATCTGCGTTTTATTGATCCGGCAATTGTTCAGTGTATGTATGGTCGTGAAGATCGTAATA<br>ATGCATGTCCGAGCCTGCGTCAGACCGGTGCAGAAGTGATTGGTTTTAGCGGTGGTCATC<br>ATTTTGGCAACGATTTCAAAAAACTGAGCACCCGTGTTGTGAGCGGTCTGGTTGCACGTC<br>TGAGTCATCAGTATAGCAGCGGTCCGGCTCCGCTG	
Sequence of synthetic gene for pTrc200_*virJ-phoA* construction	AGGAAACAGACC**ATGGCGATAAAATTGGTATTGATACTCGTATTTACACTGTTTCTCGCG**<br>**GCAGACGCTGCCTATGCG**GCTCAGGGCGATATTACTGCACCCGGCGGTGCTCGCCGTTT<br>AACGGGTGATCAGACTGCCGCTCTGCGTGATTCTCTTAGCGATAAACCTGCAAAAAATATT<br>ATTTTGCTGATTGGCGATGGGATGGGGGACTCGGAAATTACTGCCGCACGTAATTATGCC<br>GAAGGTGCGGGCGGCTTTTTTAAAGGTATAGATGCCTTACCGCTTACCGGGCAATACACT<br>CACTATGCGCTGAATAAAAAAACCGGCAAACCGGACTACGTCACCGACTCGGCTGCATCA<br>GCAACCGCCTGGTCAACCGGTGTCAAAACCTATAACGGCGCGCTGGGCGTCGATATTCA<br>CGAAAAAGATCACCCAACGATTCTGGAAATGGCAAAAGCCGCAGGTCTGGCGACCGGTA<br>ACGTTTCTACCGCAGAGTTGCAGGATGCCACGCCCGCTGCGCTGGTGGCACATGTGACC<br>TCGCGCAAATGCTACGGTCCGAGCGCGACCAGTGAAAAATGTCCGGGTAACGCTCTGGA<br>AAAAGGCGGAAAAGGATCGATTACCGAACAGCTGCTTAACGCTCGTGCCGACGTTACGCT<br>TGGCGGCGGCGCAAAAACCTTTGCTGAAACGGCAACCGCTGGTGAATGGCAGGGAAAAA<br>CGCTGCGTGAACAGGCACAGGCGCGTGGTTATCAGTTGGTGAGCGATGCTGCCTCACTG<br>AATTCGGTGACGGAAGCGAATCAGCAAAAACCCCTGCTTGGCCTGTTTGCTGACGGCAAT<br>ATGCCAGTGCGCTGGCTAGGACCGAAAGCAACGTACCACGGCAATATCGATAAGCCCGC<br>AGTCACCTGTACGCCAAATCCGCAACGTAATGACAGTGTACCAACCCTGGCGCAGATGAC<br>CGACAAAGCCATTGAATTGTTGAGTAAAAATGAGAAAGGCTTTTTCCTGCAAGTTGAAGGT<br>GCGTCAATCGATAAACAGGATCATGCTGCGAATCCTTGTGGGCAAATTGGCGAGACGGTC<br>GATCTCGATGAAGCCGTACAACGGGCGCTGGAATTCGCTAAAAAGGAGGGTAACACGCT<br>GGTCATAGTCACCGCTGATCACGCCCACGCCAGCCAGATTGTTGCGCCGGATACCAAAG<br>CTCCGGGCCTCACCCAGGCGCTAAATACCAAAGATGGCGCAGTGATGGTGATGAGTTAC<br>GGGAACTCCGAAGAGGATTCACAAGAACATACCGGCAGTCAGTTGCGTATTGCGGCGTAT<br>GGCCCGCATGCCGCCAATGTTGTTGGACTGACCGACCAGACCGATCTCTTCTACACCATG<br>AAAGCCGCTCTGGGGCTGAAATAAAAGCTTGGCTGTTTTG<br>(VirJ native signal sequence in bold)	

Table S2. Strains and plasmids

Strains and plasmids	Characteristics	Reference
**Strains**		
Agrobacterium tumefaciens		
C58	Wild-type strain containing nopaline-type Ti plasmid pTiC58	C. Baron
WT/(pTrc200)	Wildtype carrying empty plasmid pTrc200	(Groenewold *et al.*, 2019)
Δ*acvB*	Wildtype derivative, deletion of the *acvB* gene	(Groenewold *et al.*, 2019)
Δ*acvB/*(pTrc)	Δ*acvB* carrying empty plasmid pTrc200	(Groenewold *et al.*, 2019)
Δ*acvB/*(pTrc_virJ)	Complementation of Δ*acvB* with pTrc200_virJ_Strep	This work
Δ*acvB/(*pTrc_virJ_S60A)	Complementation of Δ*acvB* with pTrc200_virJS60A_Strep	This work
Δ*acvB/*(pTrc_virJ_S127A)	Complementation of Δ*acvB* with pTrc200_virJS127A_Strep	This work
Δ*acvB/*(pTrc_virJ_H223N)	Complementation of Δ*acvB* with pTrc200_virJH223N_Strep	This work
Escherichia coli		
BL21(λDE3)	F⁻ *dcm ompT hsdS*(r_B^- m_B^-) *gal* λ(DE3)	Stratagene
DH10B	F⁻ *mcrA* Δ(*mrr-hsd*RMS-*mcrBC*) Φ80d*lacZ*ΔM15 Δ*lacX*74 *deoR* *recA*1 *endA*1 *araD*139 Δ(*ara*, *leu*) 7697 *galU* *galK* λ⁻ *rpsL nupG*	Invitrogen
C600 Rif	F⁻, *thr*-1, *leu*B6, *thi*-1, *lacY1*, *gln*V44, *ton*A21, Rif^R	Lab collection RUB
**Plasmids**		
pET22b(+)Strep	pET22b(+)-derivative providing a C-terminal Strep-tag II inserted into the HindIII/XhoI site, Ap^r	(Nicke *et al.*, 2013)
pET22b(+)Strep_pelB_virJΔaa1-22	pET22b(+)Strep-derivative containing the *virJ* gene with bases 1-66 deleted	This work
pTrc200	Str^r, Spc^r, pVS1 origin, *lacI*^{q}, *trc* promoter expression vector	(Schmidt-Eisenlohr *et al.*, 1999)
pTrc200_virJ	pTrc200-derivative coding for full-length VirJ with C-terminal Strep-tag II	This work
pTrc200_virJS60A	pTrc200-derivative encoding full-length VirJS60A variant with C-terminal Strep-tag II	This work
pTrc200_virJS127A	pTrc200-derivative coding for full-length VirJS127A variant with C-terminal Strep-tag II	This work
pTrc200_virJH223N	pTrc200-derivative encoding full-length VirJH223N variant with C-terminal Strep-tag II	This work
pTrc200_Δss-*phoA*	pTrc200-derivative containing bases 100 – 1416 from *phoA* into the NcoI/HindIII site	(Groenewold *et al.*, 2019)

Strains and plasmids	Characteristics	Reference
pTrc200_native-*phoA*	pTrc200-derivative containing bases 1 – 1416 from *phoA* cloned into the NcoI/HindIII site	(Groenewold *et al.*, 2019)
pTrc200_virJ-phoA	pTrc200-derivative containing bases 1 – 66 from *virJ* and bases 102-1416 from phoA, cloned via In-Fusion® HD Cloning	This work
RP4	IncPα, Tra1, Tra2, Amp^R, Kan^R, Tet^R	(Pansegrau *et al.*, 1994)

Supplementary Figures

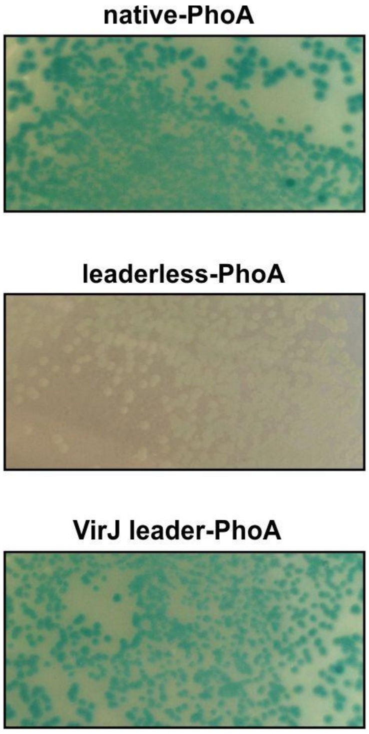

Figure S1. Periplasmic localization of VirJ in *A. tumefaciens*. *Agrobacterium* C58 strains carrying plasmids encoding PhoA with its native leader (native-PhoA), PhoA without signal sequence (leaderless-PhoA) or a fusion of the 22 amino terminal residues of VirJ with PhoA (VirJ leader-PhoA) were analyzed. Colonies of the native-PhoA containing strain (positive control) and the VirJ leader - PhoA are colored blue due to the conversion of the PhoA-specific substrate BCIP. Note that the control plates were previously published for AcvB localization (Groenewold et al., 2019) and are reused here, as experiments for both AcvB and VirJ were performed in parallel under identical conditions.

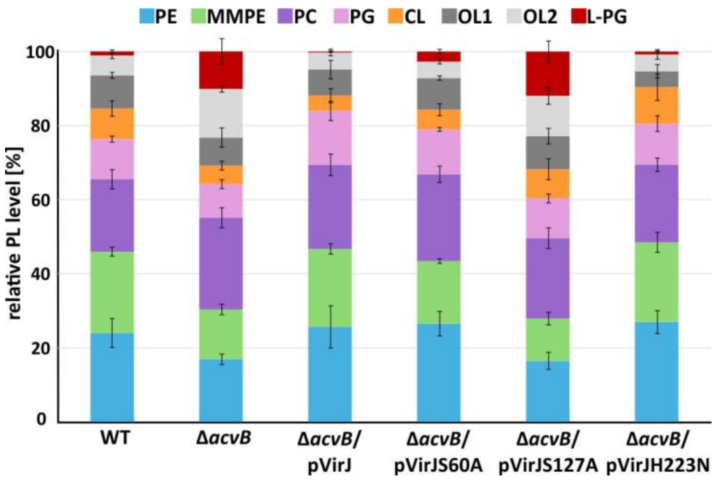

Figure S2. Relative phospholipid levels in different *A. tumefaciens* strains. Phospholipid spot intensity after molybdatophosphoric acid staining was quantified using GelQuant and normalized relative to the total lipid (dominant) content (PE, MMPE, PC, PG, CL, OL1, OL2 and L-PG). Mean values were calculated from the results of at least three independent experiments and error bars show standard errors of the means.

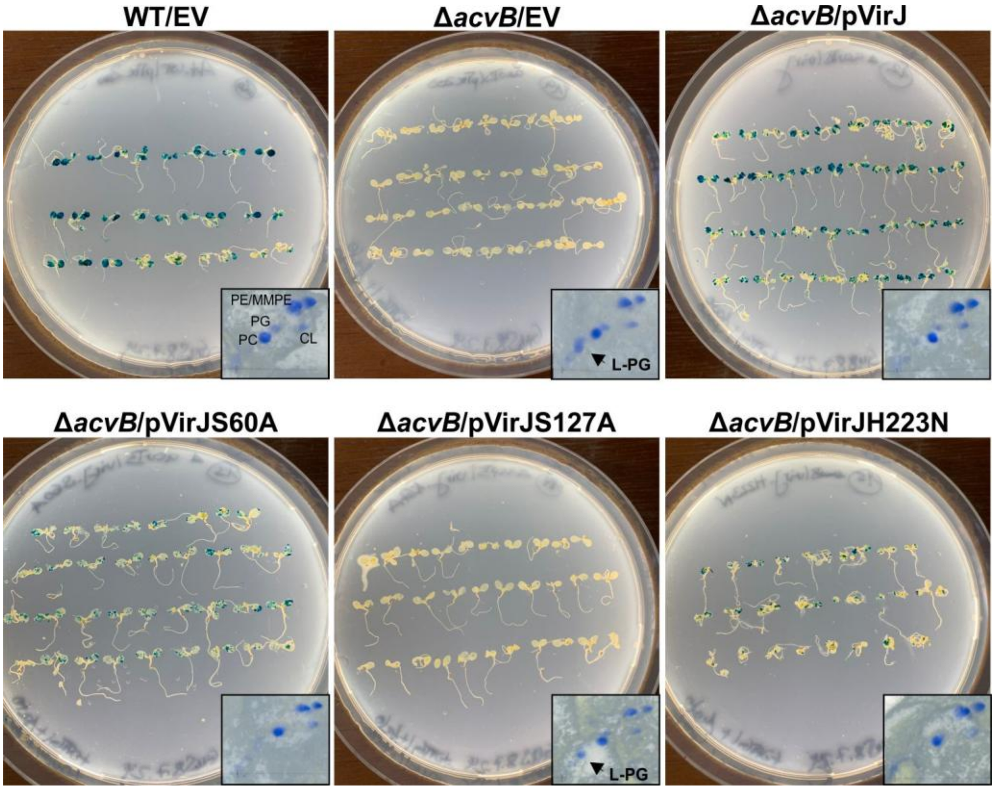

Figure S3. *Agrobacterium*-mediated transient transformation of *Arabidopsis* seedlings. Seven-day-old Arabidopsis efr-1 seedlings were infected with *Agrobacterium C58* derivatives containing pBISN1 and the corresponding pTrc200 derivatives for 3 days. The efficiency of transient GUS expression was evaluated by GUS staining. The inset shows the lipid profiles of the respective strains, highlighting the relative levels of L-PG, with increased L-PG levels indicated by an arrow. Lipids from the infection strains were isolated, separated by 2D-TLC using solvent system (2), and visualized with Molybdenum blue staining. One representative result from each strain, obtained from three independent experiments, is shown. EV: empty vector, pTrc200.

References

Groenewold, M.K., Hebecker, S., Fritz, C., Czolkoss, S., Wiesselmann, M. & Heinz, D.W. et al. (2019) Virulence of *Agrobacterium tumefaciens* requires lipid homeostasis mediated by the lysyl-phosphatidylglycerol hydrolase AcvB. *Molecular Microbiology*, 111(1), 269–286. Available from: https://doi.org/10.1111/mmi.14154.

Nicke, T., Schnitzer, T., Münch, K., Adamczack, J., Haufschildt, K. & Buchmeier, S. et al. (2013) Maturation of the cytochrome cd1 nitrite reductase NirS from *Pseudomonas aeruginosa* requires transient interactions between the three proteins NirS, NirN and NirF. *Bioscience Reports*, 33(3). Available from: https://doi.org/10.1042/BSR20130043.

Pansegrau, W., Lanka, E., Barth, P.T., Figurski, D.H., Guiney, D.G. & Haas, D. et al. (1994) Complete nucleotide sequence of Birmingham IncP alpha plasmids. Compilation and comparative analysis. *Journal of Molecular Biology*, 239(5), 623–663. Available from: https://doi.org/10.1006/jmbi.1994.1404.

Schmidt-Eisenlohr, H., Domke, N., Angerer, C., Wanner, G., Zambryski, P.C. & Baron, C. (1999) Vir proteins stabilize VirB5 and mediate its association with the T pilus of *Agrobacterium tumefaciens*. *Journal of Bacteriology*, 181(24), 7485–7492. Available from: https://doi.org/10.1128/JB.181.24.7485-7492.1999.
